# Role of the transcription factor Wor2 in biofilm formation of *Candidozyma auris*

**DOI:** 10.1128/msphere.00057-26

**Published:** 2026-04-20

**Authors:** Marine Louvet, Jizhou Li, Maialen Areitio, Danielle Brandalise, Daniel Bachmann, Alix T. Coste, Salomé LeibundGut-Landmann, Dominique Sanglard, Frederic Lamoth

**Affiliations:** 1 Institute of Microbiology, Department of Laboratory Medicine and Pathology, Lausanne University Hospital and University of Lausanne 536517, Lausanne, Switzerland; 2 Infectious Diseases Service, Department of Medicine, Lausanne University Hospital and University of Lausanne 30639, Lausanne, Switzerland; 3 Section of Immunology, Vetsuisse Faculty and Institute of Experimental Immunology, University of Zurich 27217, Zürich, Switzerland; 4 Immunology, Microbiology and Parasitology Department, Faculty of Science and Technology, University of the Basque Country (UPV/EHU) 200145 , Leioa, Spain; 5 Department of Biosciences, Faculty of Health and Life Sciences, Medical Research Council Centre for Medical Mycology at the University of Exeter, Exeter, United Kingdom; Hackensack Meridian Health Center for Discovery and Innovation, Nutley, New Jersey, USA

**Keywords:** *Candida auris*, adhesin, Ywp1, Als4112, Scf1

## Abstract

The yeast pathogen *Candidozyma* (*Candida*) *auris* can form biofilms, which contribute to its virulence and nosocomial transmission. In this study, we identified the transcription factor Wor2 as a negative regulator of biofilm formation in *C. auris*. Wor2 hyperactivation in a strain of clade IV via the use of a protein tagging strategy resulted in downregulation of two important adhesins, *SCF1* and *ALS4112*, and decreased biofilm-forming capacity. We showed that the impact on biofilm was predominantly mediated via decreased *SCF1* expression in this strain. However, results of adhesion assays on inert surfaces and human keratinocytes found relatively modest roles of Wor2 and Scf1 in this process, suggesting that their effect on biofilm formation is complex and not limited to the adhesion step. Finally, analyses of other strains from different clades identified three distinct *WOR2* genotypes, with variable *WOR2* expression levels and distinct impacts of *WOR2* deletion on biofilm formation. Notably, Wor2 negatively regulated biofilm in strains of clades I, III, and IV with distinct profiles of *SCF1*/*ALS4112* expression, while it had no impact on biofilm in a clade II strain. Taken together, this study showed that Wor2 exhibited some distinct genotypic evolution in *C. auris* resulting in clade- or strain-specific regulatory roles and pathways in biofilm formation.

**IMPORTANCE:**

*Candidozyma* (*Candida*) *auris* is a pathogenic yeast exhibiting a particular capacity for interhuman transmission via medical instruments, which was the cause of nosocomial outbreaks of candidemia. Adhesion to inert surfaces and subsequent biofilm formation is therefore important for *C. auris* propagation. This work highlights the role of the transcription factor Wor2 as a negative regulator of biofilm formation in *C. auris*. In a strain of clade IV, Wor2 was shown to downregulate two important adhesins (*SCF1* and *ALS4112*). Interestingly, Wor2 exhibited different genotypes across *C. auris* clades and strains, which were associated with distinct differential expression of *WOR2*, *ALS4112*, and *SCF1*, and possibly distinct roles in biofilm formation.

## INTRODUCTION

*Candida auris* (*Candidozyma auris*, according to the new taxonomy) is a pathogenic yeast that has been recognized as a major infectious threat because of its antifungal resistance, persistence in healthcare environments, and high potential for interhuman transmission leading to nosocomial outbreaks (1–3). Notably, *C. auris* displays resistance to standard surface disinfectants and can survive on inert surfaces for extended periods (4, 5). Like other pathogenic *Candida* species, *C. auris* forms biofilms (i.e., structured microbial communities embedded in an extracellular matrix), which contributes to its persistence, transmission, and pathogenicity (6, 7). *C. auris* demonstrated a unique ability to form biofilms on porcine skin and in synthetic sweat medium mimicking axillary skin conditions (8). Among clinical *C. auris* isolates, strong biofilm producers have been predominantly recovered as colonizing isolates (9). Moreover, biofilms on medical devices can be the source of systemic *C. auris* infections. Colonization of intravascular catheters has been associated with candidemia (10–12). *C. auris* also demonstrated a high propensity to colonize urine via urinary catheters and was recently recognized as an important urinary pathogen (13).

Mechanisms of biofilm formation have been extensively studied in *Candida albicans* but remain poorly elucidated in *C. auris* (14, 15). Adhesion is a first critical step in the process of biofilm formation (14). Some adhesins have been shown to play a crucial role in *C. auris* adhesion and biofilm formation, such as the agglutinin-like sequence adhesin Als4112 (formerly referred to as Als4) and the *C. auris*-specific surface colonization factor Scf1 (16–19). Als4112 is responsible for the aggregative phenotype with increased cell-to-cell adherence and biofilm-forming capacity (16, 18, 20). It has also been shown to be crucial for skin colonization via adhesion to keratinocytes and interactions with extracellular matrix proteins (19). Scf1 is required for biofilm formation on inert surfaces, intravascular catheters, and skin and plays a role in *C. auris* virulence (17). We have recently shown that the zinc cluster transcription factor (ZCF) Ume6 was involved in biofilm formation of *C. auris* via Scf1 (21).

In this work, we demonstrated that the white-opaque regulator 2 (Wor2), another ZCF, is a repressor of biofilm formation in *C. auris*, partly via downregulation of *ALS4112* and *SCF1*, with some distinct specificities across strains and clades.

## RESULTS

### Wor2 is a negative regulator of biofilm formation

To investigate the role of ZCFs in *C. auris*, we have constructed a mutant library, in which ZCFs genes were put under the control of a constitutive promoter (*P_ADH1_*) with the addition of a 3xHA C-terminal tag, as previously described (21, 22). The constructs were cloned in the *C. auris* neutral site (*CauNI*) of strain IV.1 (a clinical isolate of clade IV), which resulted in overexpression and hyperactivation of the ZCF, as previously shown (21, 22). We observed that hyperactivation of the gene SBP28_003124 (corresponding to B9J08_002136 in strain B8441) resulted in altered biofilm formation. This gene was found to be the ortholog of *WOR2* in *Candida albicans* (sharing 27.3% identity). This hyperactivated strain was therefore named *WOR2^HA^*. To further investigate the role of Wor2 in biofilm formation, we also generated a *WOR2* deletion strain (*wor2*Δ) in IV.1.

**Fig 1:**
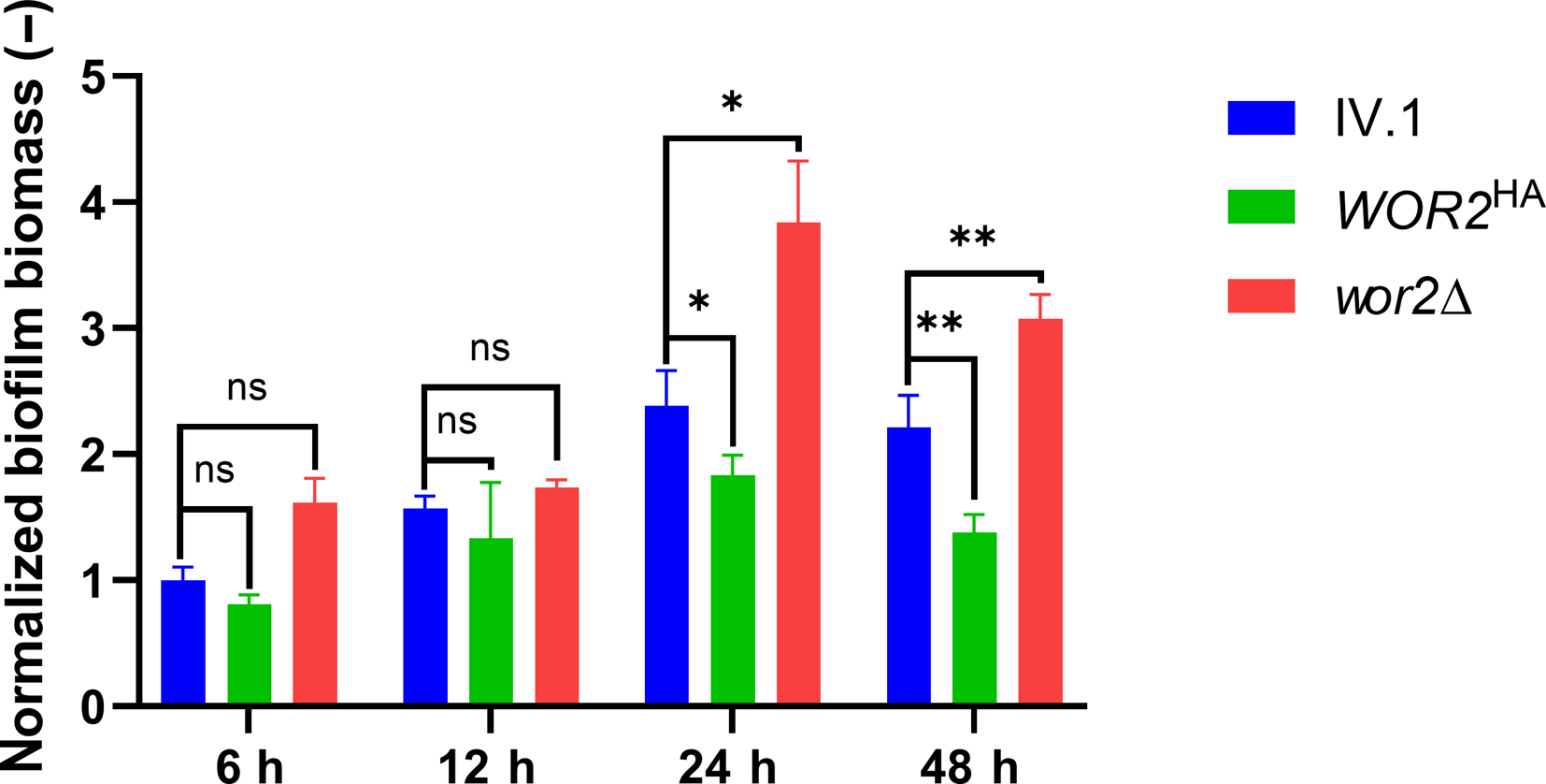
Relative quantification of biofilm mass of *WOR2^HA^* and *wor2*∆ compared to IV.1 (background strain) at different time points. Relative biofilm biomass (represented by absorbance at 590 nm) was measured by crystal violet assay after 6 h, 12 h, 24 h, and 48 h of incubation and expressed as fold change compared to the background IV.1 strain at 6 h. Bars represent means with standard deviations of biological triplicates and technical duplicates. Statistical analysis was performed using unpaired *t*-test with significant *P*-value defined as * ≤ 0.05, ** ≤ 0.01. ns, not significant (*P*-value > 0.05).

Quantification of biofilm mass by crystal violet assay at different time points showed that the *WOR2^HA^* and *wor2*Δ strains produce significantly less and more biofilm, respectively, at 24 h and 48 h compared to their parental strain (Fig. 1). We concluded that Wor2 is a repressor of biofilm formation in *C. auris*. Of note, no growth defect or morphological alteration was observed for any of the mutant strains (*WOR2^HA^* and *wor2*Δ) (Fig. S1). Because Wor2 has been associated with the white-opaque switch in *C. albicans* (23), we also tested morphogenetic aspects of the colonies at different temperatures with addition of phloxine B in agar plates (to reveal color switch). We did not find any phenotypic alteration among Wor2 mutants in these conditions (Fig. S2).

### Wor2 downregulates the adhesins *SCF1* and *ALS4112*, but its role in biofilm does not exclusively rely on this pathway

To investigate the pathways of Wor2 in biofilm formation, we performed transcriptomic analyses (RNA sequencing) to identify genes that were up- or downregulated in *WOR2^HA^* compared to IV.1. This analysis confirmed the effective overexpression of *WOR2* in *WOR2^HA^* (166.7-fold compared to IV.1, File S1). A total of 60 and 31 genes were significantly (i.e., ≥2-fold expression change and *P* < 0.05) up- and downregulated in *WOR2^HA^*, respectively (File S1). Gene ontology (GO) term analysis of upregulated genes showed enrichment in aspartyl-protease activity (e.g., candidapepsins, yapsins), metal ion transport-assimilation, and cell wall remodeling, while downregulated genes were enriched in products mainly located in the extracellular region or plasma membrane (Fig. S3; File S1). Considering the effect of Wor2 on biofilm formation, we focused on genes with significant up- or downregulation in *WOR2^HA^* and with a known or putative role in this process in *C. auris* or other *Candida* spp. These genes include *YWP1* (SBP28_000534, B9J08_003550), *ALS4112* (SBP28_005090, B9J08_004112), and *SCF1* (SBP28_003606, B9J08_001458). Ywp1 is a glycosylphosphatidylinositol (GPI) anchor protein of the cell wall, whose deletion has been associated with increased adhesion and biofilm formation in *C. albicans* (24, 25). This gene was upregulated in *WOR2^HA^* (2.5-fold compared to IV.1) in our transcriptomic data set. Als4112 and Scf1 are two adhesins playing a key role in biofilm formation of *C. auris* (16–21). These genes were downregulated in *WOR2^HA^* (−2.3-fold and −2-fold, respectively).

**Fig 2:**
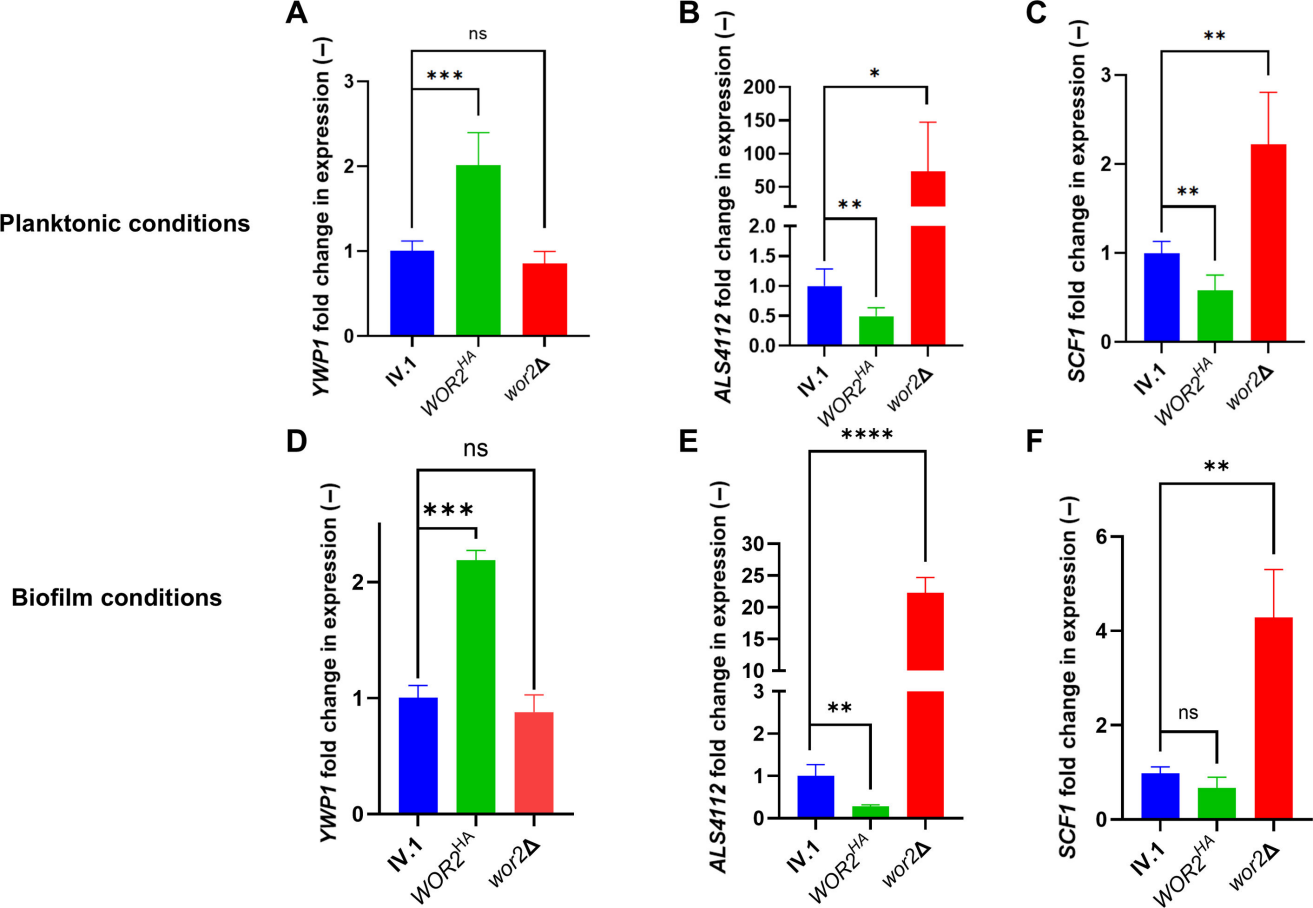
Relative expression of *YWP1*, *ALS4112,* and *SCF1* in *WOR2^HA^* and *wor2*∆ compared to IV.1 (background strain) in planktonic (**A–C**) and biofilm (**D–F**) conditions. Results are expressed as fold change compared to the background IV.1 strain. Bars represent means with standard deviations of biological triplicates and technical triplicates. Statistical analysis was performed using unpaired *t*-test with significant *P*-value defined as * ≤ 0.05, ** ≤ 0.01, *** ≤ 0.001, **** ≤ 0.0001. ns, not significant (*P*-value > 0.05).

To confirm the regulatory role of Wor2 on these three genes (*YWP1*, *ALS4112,* and *SCF1*), we measured their expression by real-time reverse transcription PCR (RT-PCR) in the *WOR2^HA^* and *wor2*Δ backgrounds under both planktonic and biofilm conditions (Fig. 2). This experiment confirmed that Wor2 positively regulates *YWP1* (showing increased expression in *WOR2^HA^*) and negatively regulates *ALS4112* and *SCF1* (showing decreased expression in *WOR2^HA^*). Deletion of *WOR2* resulted in significant overexpression of *ALS4112* and *SCF1*, while *YWP1* expression was unchanged. No relevant differences of expression profiles were observed between planktonic and biofilm conditions, except that the decreased expression of *SCF1* in *WOR2^HA^* did not reach statistical significance in biofilm conditions.

**Fig 3:**
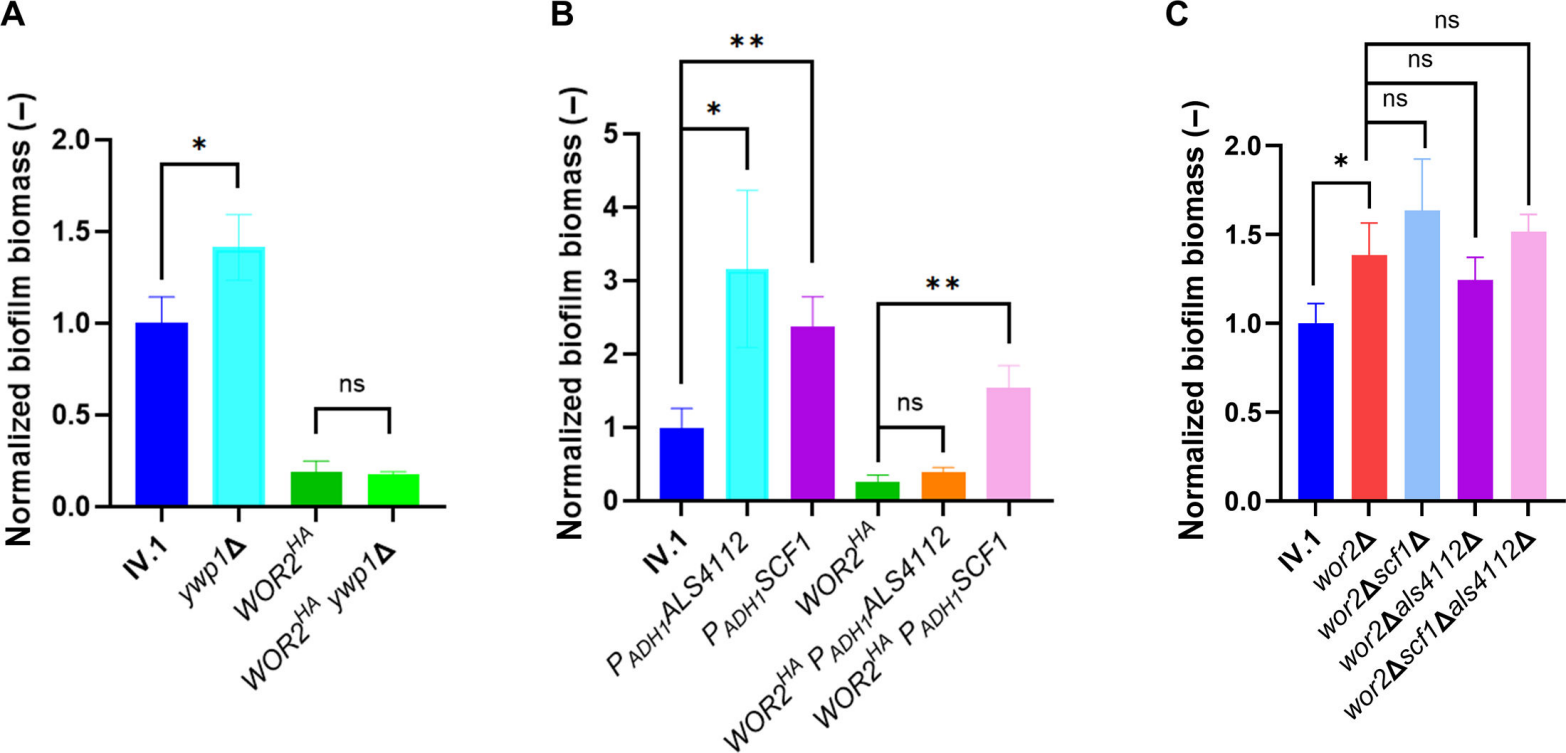
Relative quantification of biofilm mass of different mutant strains disrupting the Wor2-Ywp1 (**A**) and Wor2-Als4112/Scf1 axes (**B and C**). Relative biofilm biomass (represented by absorbance at 590 nm) was measured by crystal violet assay after 24 h of incubation and expressed as fold change compared to the background IV.1 strain. Bars represent means with standard deviations of biological duplicates and technical triplicates. Statistical analysis was performed using unpaired *t*-test with significant *P*-value defined as * ≤ 0.05, ** ≤ 0.01, *** ≤ 0.001, **** ≤ 0.0001. ns, not significant (*P*-value > 0.05).

To explore the role of Ywp1 in biofilm formation, we generated deletion strains in the IV.1 and *WOR2^HA^* backgrounds (*ywp1*Δ and *WOR2^HA^ywp1*Δ, respectively). A modest but significant increase in biofilm mass was observed following *YWP1* deletion in IV.1 but not in *WOR2^HA^* (Fig. 3A). These results suggest that Ywp1 has an inhibitory role on biofilm formation but is not the main downstream effector of *WOR2^HA^* in this process.

We then investigated the link between Wor2 and the downregulated *ALS4112* and *SCF1* genes. For this purpose, we generated overexpressing strains in which the native promoter of the target gene was substituted by the *ADH1* promoter (P*_ADH1_*) in the IV.1 and *WOR2^HA^* backgrounds. Overexpression of these genes was confirmed by RT-PCR in all mutant strains (Fig. S4). As expected, overexpression of *ALS4112* and *SCF1* in the IV.1 background (*P_ADH1_ALS4112* and *P_ADH1_SCF1*) resulted in increased biofilm capacity (Fig. 3B), which confirmed the role of these genes in biofilm formation. In the *WOR2^HA^* background, overexpression of *SCF1* (*WOR2^HA^P_ADH1_SCF1* strain) could restore biofilm capacity to a level comparable to that of IV.1, which was not the case following overexpression of *ALS4112* (*WOR2^HA^P_ADH1_ALS4112* strain) (Fig. 3B). These results suggest that the repressive effect of Wor2 on biofilm is predominantly mediated by Scf1.

As a next step, we deleted *SCF1* and/or *ALS4112* in the *wor2*Δ background (*wor2*Δ*scf1*Δ, *wor2*Δ*als4112*Δ, *wor2*Δ*scf1*Δ*als4112*Δ strains). We observed that suppression of these adhesins did not significantly impact the biofilm-forming capacity of *wor2*Δ (Fig. 3C). This observation suggests that the role of wor2 on biofilm is not exclusively mediated via Scf1 and Als4112, and that other adhesins may have a compensatory effect.

### Wor2 decreases adhesion of *C. auris* to N/TERT-1 human keratinocytes, but not to polystyrene microspheres

**Fig 4:**
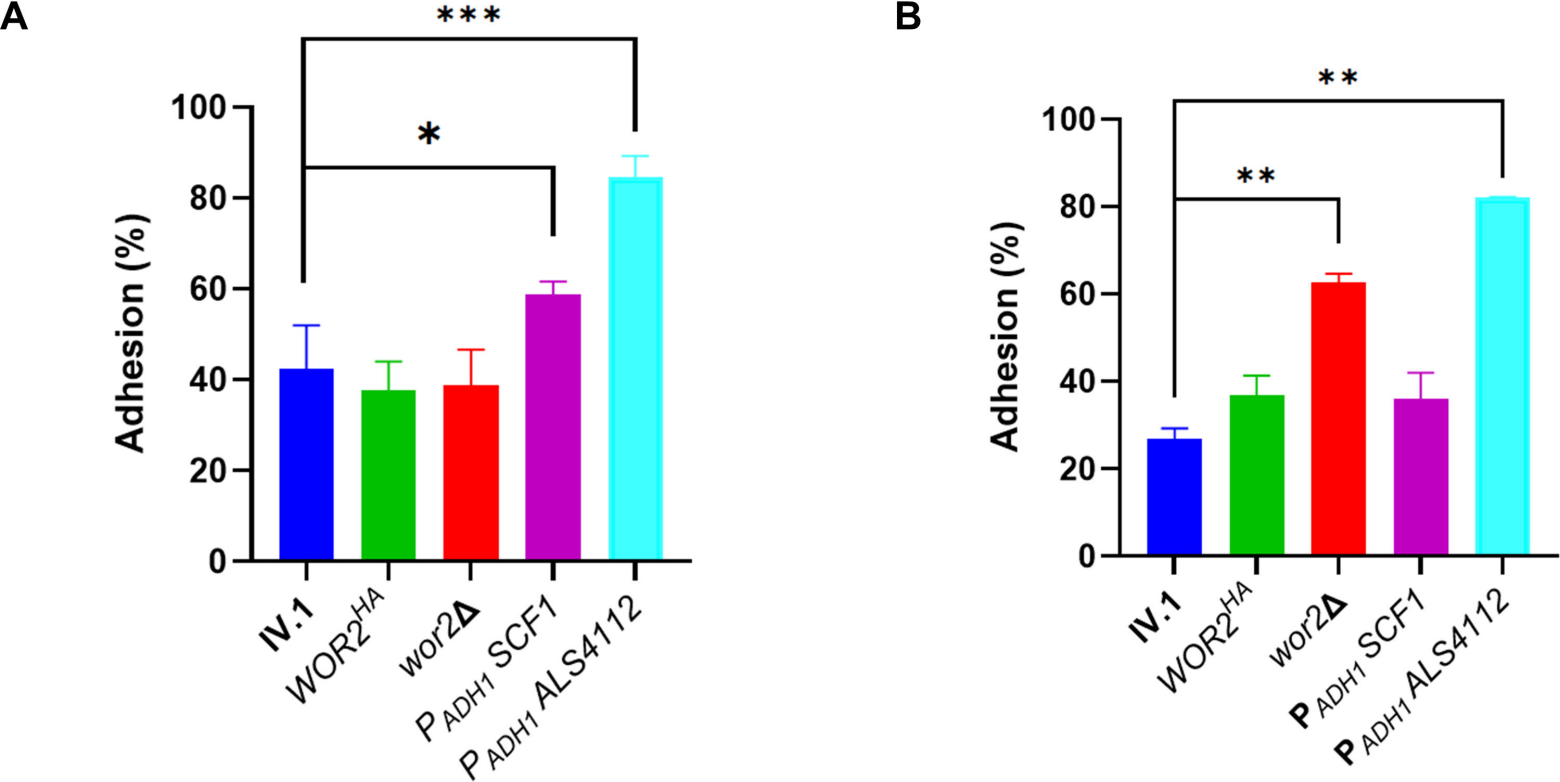
Adhesion assays on polystyrene microspheres (**A**) and N/TERT-1 human keratinocytes (**B**). Adhesion of the different strains on polystyrene microspheres was measured by flow cytometry. Results are expressed as a percentage of adherent cells for the different strains. Bars represent means with standard deviation of biological duplicates and technical triplicates. Adhesion of the different strains to N/TERT-1 keratinocytes was expressed as a percentage of adherent cells. Bars represent means with standard deviation of biological duplicates and technical duplicates. Statistical analysis was performed using unpaired *t*-test with significant *P*-value defined as * ≤ 0.05, ** ≤ 0.01, *** ≤ 0.001, **** ≤ 0.0001. ns, not significant (*P*-value > 0.05)

To further investigate the role of Wor2 in adhesion, we used flow cytometry to measure adhesion of yeast cells to inert surfaces using polystyrene microsphere beads (Fig. 4A). No significant change of adhesion percentage was observed in *WOR2^HA^* and *wor2*Δ. As expected, strains overexpressing the adhesins *ALS4112* and *SCF1* exhibited a significant increase in adhesion compared to their background IV.1. Adhesion of the mutant cells to human keratinocytes (N/TERT-1) was also assessed (Fig. 4B). *WOR2* deletion resulted in significantly increased adhesion, while no significant change was observed in *WOR2^HA^*. Regarding the adhesins, only *ALS4112* (and not *SCF1*) overexpression resulted in a significant increase in adhesion. Overall, these results show a relatively modest effect of Wor2 and Scf1 on adhesion to inert surfaces and human keratinocytes, which relies mainly on Als4112. They suggest that Wor2 may impact biofilm formation through steps other than adhesion (e.g., maturation or dispersion) and/or via other downstream targets.

### Clade- and strain-specific *WOR2* genotypic variations impact gene expression but not biofilm formation

**Fig 5:**
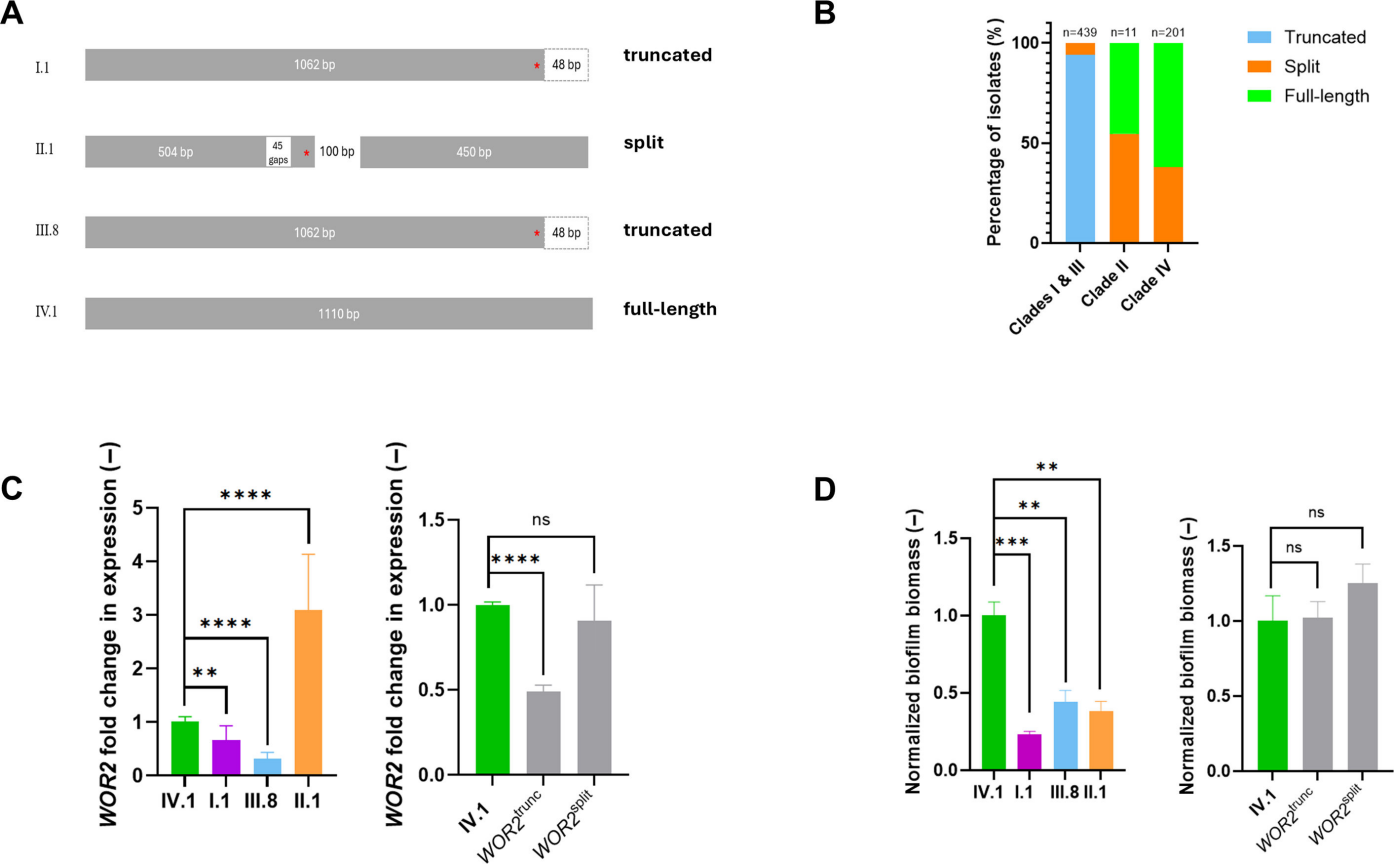
Analysis of the different *WOR2* genotypes across *C. auris* strains from different clades. (**A**) Schematic representation of the four distinct *WOR2* genotypes for four strains representative of four different clades (I to IV). Compared to the *WOR2* sequence of strain I.1 (full-length genotype), the sequence of strains I.1 and III.8 displays a truncation of 48 bp at the C-terminal locus (truncated genotype), while the *WOR2* gene of strain II.1 has an intercalated non-coding sequence of 100 bp and a gap of 45 bp (split genotype). Red stars represent stop codons. (**B**) Screening of *WOR2* genotypes across 651 *C. auris* clinical isolates from the four different main clades (I to IV). Data showed that the truncated genotype was essentially observed in clades I and III, while the strains from clades II and IV exhibited a mix of the full-length and split genotypes. The number (N) of strains analyzed for each group is indicated at the top of the bars. (**C**) Relative expression of *WOR2* according to different *WOR2* genotypes. Clinical isolates are represented in the left panel. Mutant strains, in which the full-length genotype has been substituted by the truncated or split genotypes in the IV.1 background (*WOR2*^trunc^ and *WOR2*^split^, respectively), are represented in the right panel. Results are expressed as fold change compared to IV.1. Bars represent means with standard deviations of biological triplicates and technical duplicates. Statistical analysis was performed using unpaired *t*-test with significant *P*-value defined as * ≤ 0.05, ** ≤ 0.01, *** ≤ 0.001, **** ≤ 0.0001. ns, not significant (*P*-value > 0.05). (**D**) Relative biofilm biomass of the different *WOR2* genotypes (represented by absorbance at 590 nm) was measured by crystal violet assay after 24 h of incubation and expressed as fold change compared to the background IV.1 strain. Clinical isolates are represented in the left panel. Mutant strains, in which the full-length genotype has been substituted by the truncated or split genotypes in the IV.1 background (*WOR2*^trunc^ and *WOR2*^split^, respectively), are represented in the right panel. Bars represent means with standard deviations of biological triplicates and technical duplicates. Statistical analysis was performed using unpaired *t*-test with significant P-value defined as * ≤ 0.05, ** ≤ 0.01, *** ≤ 0.001, **** ≤ 0.0001. ns, not significant (*P*-value > 0.05).

As a next step, we investigated the potential differential role of Wor2 in biofilm formation across distinct *C. auris* clades or strains. By alignment of the *WOR2* open reading frame (ORF) of four isolates from distinct clades (I.1, II.1, III.8, and IV.1), we observed some nucleotide variations (Fig. 5A). Compared to *WOR2* from IV.1 (used for genetic manipulations in the present study, and further referred to as the “full-length” genotype), strains I.1 and III.8 exhibited a C-terminal truncation of 48 bp (further referred to as “truncated” genotype). In contrast, strain II.1 displayed a gap of 45 bp (corresponding to amino-acid position 168 to 183 in IV.1) and a stop codon at position 192 with a 100 bp intercalating sequence between the next start codon (position 213) resulting in two open reading frames (ORFs) of 504 and 450 bp, respectively (further referred to as “split” genotype). A genotypic analysis of 651 clinical isolates from different clades revealed that strains from clades I and III exhibited mainly (94.5%) the truncated genotype, while strains from clades II and IV exhibited a mix of full-length and split genotypes (Fig. 5B).

We next analyzed the impact of these genetic variants on *WOR2* expression and biofilm formation. When compared to the IV.1 strain (full-length genotype), the I.1 and III.8 strains (truncated genotype) displayed lower *WOR2* expression, while the II.1 strain (split genotype) displayed increased *WOR2* expression (Fig. 5C). These results did not correlate with experiments of biofilm mass quantification, which showed more biofilm in IV.1 compared to I.1, II.1, and III.8 (Fig. 5D), suggesting that other (Wor2-independent) mechanisms are involved in biofilm formation of these clinical isolates.

To assess the actual impact of the different genotypes on *WOR2* expression and biofilm formation, we generated strains expressing the truncated and split genotypes in the IV.1 background (*WOR2*^trunc^ and *WOR2*^split^ strains, respectively). This experiment confirmed the lower *WOR2* expression associated with the truncated genotype, while no significant change was observed between the full-length and split genotypes (Fig. 5C). However, no significant changes in biofilm biomass were observed between the different mutant strains (Fig. 5D). Taken together, these analyses showed that, despite the existence of distinct clade- or strain-specific *WOR2* genotypes resulting in differential *WOR2* expression levels, these variations do not translate into significant impact on biofilm formation.

Of note, because the split genotype (strains II.1 and *WOR2*^split^) consists of two distinct ORFs, we also measured expression of the proximal ORF, which was comparable to that of the distal ORF (shown in the above experiments, Fig. S5).

### *WOR2* deletion has a distinct impact on *ALS4112*/*SCF1* expression and biofilm-forming capacity across *C. auris* strains

**Fig 6:**
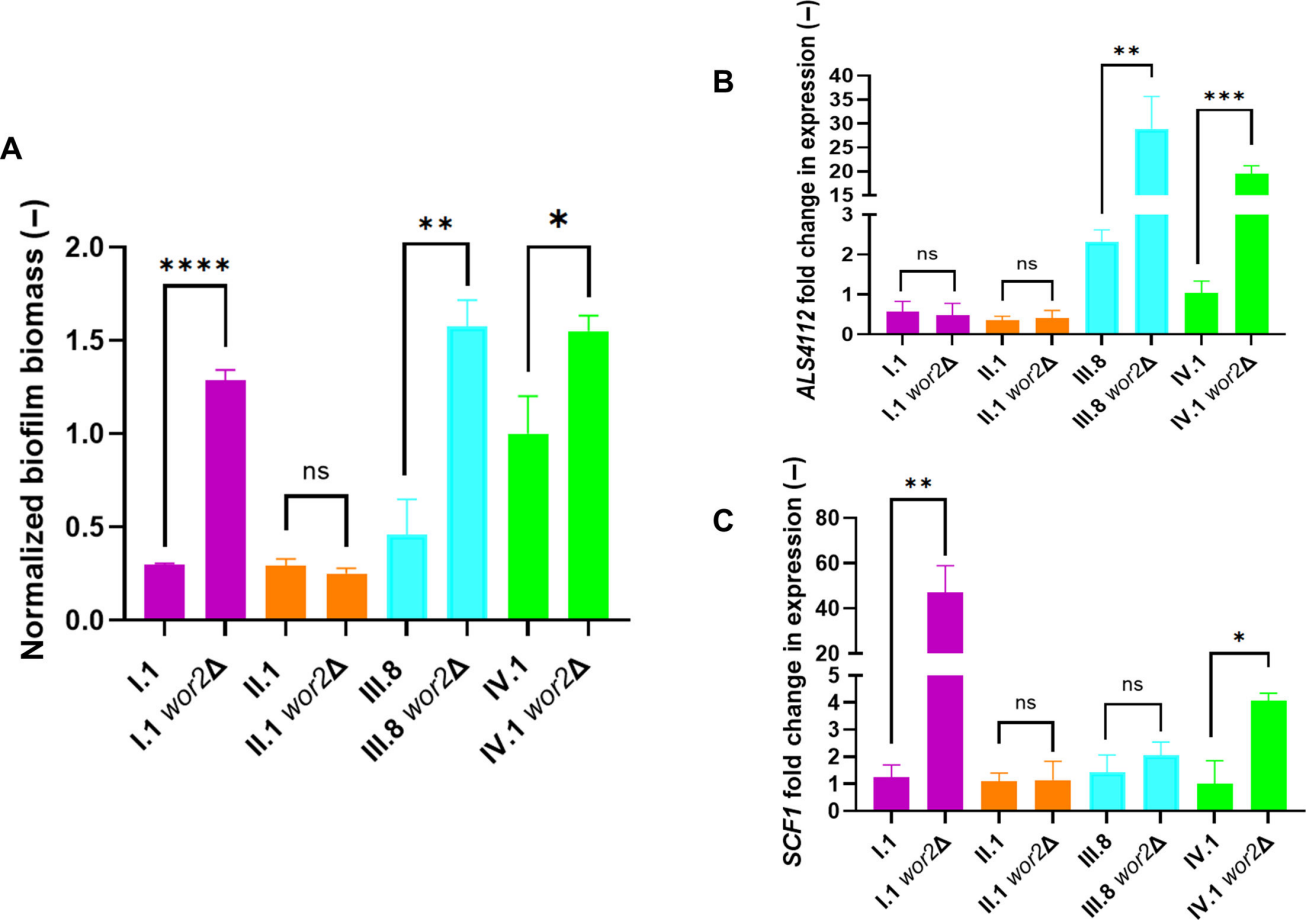
Analyses of *WOR2* deletion in *C. auris* strains from different clades. (**A**) Relative biofilm biomass (represented by absorbance at 590 nm) was measured by crystal violet assay after 24 h of incubation and expressed as fold change compared to the IV.1 strain. Bars represent means with standard deviations of biological duplicates and technical triplicates. Statistical analysis was performed using unpaired *t*-test with significant P-value defined as * ≤ 0.05, ** ≤ 0.01, *** ≤ 0.001, **** ≤ 0.0001. ns, not significant (*P*-value > 0.05). (**B**) Relative expression of *ALS4112*. Results are expressed as fold change compared to the IV.1 strain. Bars represent means with standard deviations of biological triplicates and technical triplicates. Statistical analysis was performed using unpaired *t*-test with significant *P*-value defined as * ≤ 0.05, ** ≤ 0.01, *** ≤ 0.001, **** ≤ 0.0001. (**C**) Relative expression of *SCF1*. Results are expressed as fold change compared to the IV.1 strain. Bars represent means with standard deviations of biological triplicates and technical triplicates. Statistical analysis was performed using unpaired *t*-test with significant *P*-value defined as * ≤ 0.05, ** ≤ 0.01, *** ≤ 0.001, **** ≤ 0.0001.

Finally, we wanted to assess the impact of *WOR2* deletion on biofilm formation in strains from different clades and with different *WOR2* genotypes. We therefore generated *WOR2* deletion strains in clades I, II, and III (I.1 *wor2*Δ, II.1 *wor2*Δ, and III.8 *wor2*Δ strains, respectively) and compared them to IV.1 *wor2*Δ. The impact of *WOR2* deletion was stronger in strains of clades I and III (truncated genotype, about 3.5- to 4-fold increase of biofilm mass) compared to IV.1 (about 1.5-fold increase), while no effect was observed in the strain of clade II (split genotype) (Fig. 6A). Expression of *ALS4112* and *SCF1* was also measured in these strains (Fig. 6B and C). *WOR2* deletion in III.8 resulted in a significant overexpression of *ALS4112*, but not *SCF1*. On the contrary, only *SCF1* (and not *ALS4112*) was significantly overexpressed in I.1 *wor2*Δ compared to its parental strain. In agreement with our previous experiments (Fig. 2B and C), both *ALS4112* and *SCF1* were overexpressed in IV.1 *wor2*Δ compared to IV.1. Finally, no change of *ALS4112* and *SCF1* expression was observed in II.1 *wor2*Δ compared to II.1, which is consistent with the absence of impact on biofilm formation following *WOR2* deletion in this strain. This observation suggests that the Wor2 split genotype (present in II.1) may have a functional defect.

**Fig 7:**
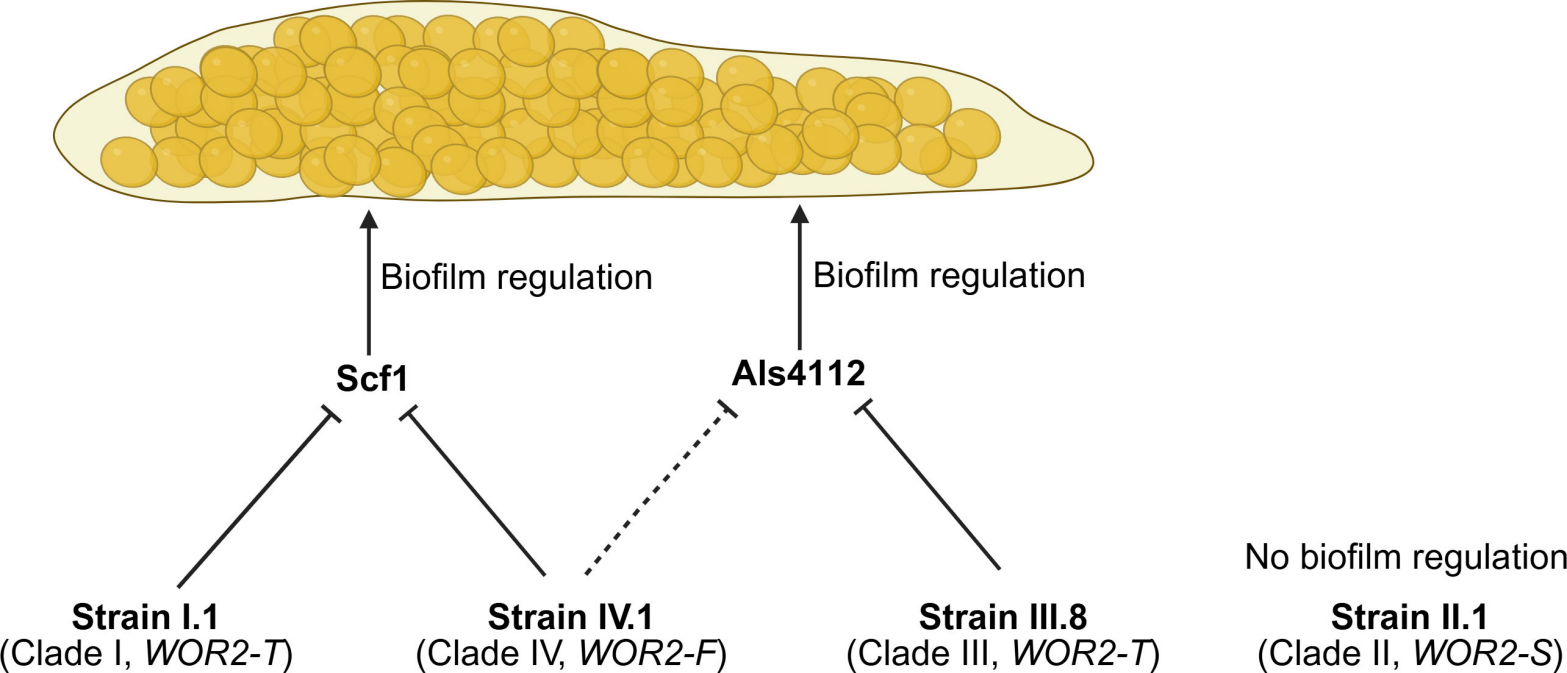
Schematic representation of the roles and pathways of Wor2 in biofilm formation in different *C. auris* strains from different clades. The transcription factor Wor2 exhibits three distinct genotypes, *WOR2-F* (full-length), *WOR2-T* (truncated), and *WOR2-S* (split), with some clade or strain specificity. Wor2 has distinct roles (via downregulation of *SCF1* and/or *ALS4112*) in biofilm formation. Image generated with BioRender.

Taken together, these results suggest that Wor2 may have distinct roles and pathways in controlling biofilm formation across different *C. auris* clades and strains, as illustrated in Fig. 7.

## DISCUSSION

In this study, we investigated the role and pathways of the ZCF Wor2 in *C. auris* biofilm formation. In *C. albicans* and other related species (*C. dubliniensis*, *C. tropicalis*), Wor2 is a regulator of the white-opaque switching, a morphological change driven by an epigenetic phenomenon also involving other transcription regulators, such as Wor1, Wor3, Czf1, Efg1, and Ahr1 (23, 26). Several triggers of white-opaque switching have been identified, such as temperature changes, low pH, anaerobic conditions, high CO_2_, and N-acetylglucosamine (27). Previous studies showed that *C. auris* was also capable of morphogenetic switching from white to pink or white to brown (28, 29). We did not observe any morphological alteration following Wor2 hyperactivation or deletion in *C. auris*, but we found a significant impact on biofilm formation, which has not been reported in *C. albicans*. Transcriptional regulatory circuits controlling morphogenetic pathways, such as biofilm formation and white-opaque switching, were shown to be interconnected in *C. albicans* (30). This finding is therefore not surprising. Moreover, transcriptional rewiring (i.e., divergence of regulatory pathways) is a well-known evolutionary process in *Candida* spp. (31, 32), which may explain these distinct Wor2 functions between *C. albicans* and *C. auris*.

To decipher the regulatory circuitry of Wor2 in biofilm formation of *C. auris*, we tried to identify its downstream targets. Our transcriptomic analysis showed that Wor2 negatively regulates the expression of two adhesins, *SCF1* and *ALS4112*, which are known to play a major role in *C. auris* biofilms (16–19). *SCF1* was previously found to be positively regulated by the ZCF Ume6 (21), while the regulator of *ALS4112* has not yet been identified. Interestingly, constitutive expression of *SCF1* (via the *ADH1* promoter) could reverse the repressive effect of Wor2 hyperactivation on biofilm formation in a strain of clade IV, which was not the case following overexpression of *ALS4112*. These results suggest that Wor2 regulates biofilm formation mainly via Scf1 in this strain. On the contrary, deletion of *SCF1* and/or *ALS4112* in the wor2Δ background did not alter the increased biofilm-forming capacity of this strain, suggesting that Wor2 may control other important pathways in biofilm formation and/or that other adhesins may compensate for the loss of Scf1 and Als4112. Finally, our adhesion assays showed a relatively modest role of Wor2 and Scf1 in adhesion, which was mainly triggered by *ALS4112* overexpression. These results suggest that Als4112 is the adhesin playing the key role in this first step of biofilm formation, while Wor2, via Scf1 and possibly other targets, is mainly involved in subsequent steps of biofilm development.

Biofilm formation is a complex phenomenon, starting with adhesion and involving multiple subsequent steps, such as cell proliferation, morphogenetic changes (e.g., filamentation), production of extracellular polymeric substances, and maturation of multilayered network of polymorphic cells (15). Among other downregulated genes in *WOR2*^HA^, we found an enrichment in gene products of the extracellular region, which may suggest some role in the development of the extracellular matrix. Among upregulated genes, we found an enrichment of the secreted aspartyl proteases (SAPs) family, such as candidapepsins and yapsins. Interestingly, SAPs have been involved in biofilm formation in *C. albicans* and other pathogenic *Candida* species (33–37). Notably, some of them act on specific extracellular signaling proteins, such as Msb2, which activates the MAP kinase pathway (38). However, their link to Wor2 remains elusive in *C. auris*. Because Wor2 inhibits biofilm formation in *C. auris*, this upregulation of multiple SAPs in *WOR2^HA^* was rather interpreted as a non-specific compensatory effect. We also observed upregulation of genes involved in cell wall remodeling and focused our attention on Ywp1, a GPI anchor protein, which was shown to have an anti-adhesive effect in *C. albicans* (24, 25). Although we could demonstrate a negative impact of Ywp1 in biofilm formation of *C. auris*, we found that this effect was independent from Wor2. The mechanisms by which Ywp1 represses biofilm formation in *C. auris* are still unknown and would deserve further investigations, such as analyses of its impact on the expression of different adhesins.

Finally, we observed three distinct *WOR2* genotypes exhibiting some clade specificity. It should be noted that these observations resulted from deposited whole-genome sequencing data of a public repository (FungiDB), which may contain some errors. However, these results were consistent with our own *WOR2* sequencing data of different representative strains from each clade. We could demonstrate that these genotypes were associated with distinct levels of *WOR2* expression, which were not sufficient to translate into significant differences in biofilm-forming capacity. However, *WOR2* deletion resulted in a variable impact on biofilm formation, as well as distinct profiles of *ALS4112* and *SCF1* expression, across strains from different clades. These results suggest that the roles and pathways of Wor2 in biofilm formation may be clade- or strain-specific, as illustrated by the schematic representation in Fig. 7. In the strain of clade I, *WOR2* deletion resulted in a significant increase of biofilm formation in association with an increased expression of *SCF1* only (not *ALS4112*), which supports the predominant role of Scf1 in this pathway, in accordance with our previous observations in strain IV.1 (clade IV). On the contrary, only *ALS4112* (and not *SCF1*) was overexpressed in the *WOR2* deletion strain of clade III, which also resulted in significant increase of biofilm formation. This strain exhibited a higher basal level of *ALS4112* expression, which is consistent with previous reports showing increased *ALS4112* gene copy numbers in strains of clade III associated with a particular aggregative phenotype (16). Finally, *WOR2* exhibited a high level of basal expression in the strain of clade II, but its deletion had no impact on *SCF1*/*ALS4112* expression and biofilm formation, which suggests that the split genotype (present in this strain) consisting of two distinct ORFs could be nonfunctional. Interestingly, the split genotype was predominant in strains of clade II but was also observed in strains of clade IV and a few strains of clades I or III (Fig. 5B). It is therefore possible that *C. auris* has developed some mechanism to facilitate Wor2 splitting as a means to turn it off. We did *WOR2* deletion in one strain representative of each clade and each genotype. However, *WOR2* deletion should be performed in a larger collection of isolates to check for reproducibility of these results and their clade or genotype specificity.

We also looked for *WOR2* orthologs in closely related species of *C. auris* in the *Metschnikowiaceae* clade. We found that the gene was present in *Candida haemulonii* (*CXQ85_002622*; 55.6% identity), *Candida duobushaemulonii* (*CXQ87_003055*; 46.5% identity), and *Candida pseudohaemulonii* (*C7M61_004473*; 42% identity). These observations suggest that *C. auris WOR2* may have followed some divergent evolution compared to other members of this clade.

In conclusion, we demonstrated that Wor2 is a negative regulator of biofilm formation in *C. auris* but with possible distinct roles and pathways across clades and strains. Whether or not these differential Wor2 roles and pathways are clade-specific or rather strain-specific should be confirmed in larger strain collections. Moreover, the pathways of Wor2 in controlling biofilm formation do not seem to be limited to its negative control on these two important adhesins but may involve other steps of biofilm formation (e.g., extracellular matrix), which would deserve further investigation.

## MATERIALS AND METHODS

### Plasmids and strains

Plasmids pDS2020 containing the *NatR* cassette (nourseothricin resistance), pCdOpt-BMX containing the *BleMX* cassette (zeocin resistance), and pYM70 containing the *HygR* cassette (hygromycin resistance) were used for the construction of the deletion strains (39–41). Plasmid pjli8, constructed from Clp-p*ACT1*-3xFLAG-MNase-SV40-*CYC-SAT1* and containing the nourseothricin resistance cassette *SAT1* and the *C. auris* neutral site *CauNI*, was used for the construction of the hyperactivated Wor2 strain (*WOR2^HA^*) (22). *Escherichia coli* DH5α was used for plasmid construction and amplification. Plasmids were extracted using the Plasmid Mini Kit (Qiagen). Primers used in this study are listed in Table S1. The *C. auris* isolate IV.1 (clade IV, LMDM 1219) was used as a source for DNA amplification and as a background strain for genetic transformations (42). *WOR2* deletion was also performed in other clinical isolates (I.1, II.2, III.8). Yeast extract-peptone-dextrose (YEPD), containing bactopeptone 20 g/L, yeast extract 10 g/L, and glucose 20 g/L, with or without agar 20 g/L, was used as culture medium. All cultures were incubated at 37°C on solid YEPD agar plates or in liquid YEPD under constant agitation (220 rpm). A complete list of the strains (clinical isolates and mutants) used in this study is provided in Table 1.

**TABLE 1:** List of the strains used for this study

Strain name	Genetic modification	Description	Reference
I.1 (B8441/AR387)	Clinical strain	Clinical strain from clade I	(43)
II.1	Clinical strain	Clinical strain from clade II	(44)
III.8	Clinical strain	Clinical strain from clade III	(45)
IV.1	Clinical strain	Clinical isolate from clade IV	(42)
*WOR2^HA^*	IV.1 *WOR2^HA^*::*SAT1*	*WOR2* hyperactivation in IV.1	This study
*wor2*∆	IV.1 *wor2*Δ::*HYGR*	*WOR2* deletion in IV.1	This study
*P_ADH1_ALS4112*	IV.1 *P_ADH1_ALS4112*::*BleMX*	*ALS4112* overexpression in IV.1	This study
*WOR2^HA^ P_ADH1_ALS4112*	IV.1 *WOR2^HA^*::*SAT1 P_ADH1_ALS4112*::*BleMX*	*ALS4112* overexpression in *WOR2^HA^* background in IV.1	This study
*P_ADH1_SCF1*	IV.1 *P_ADH1_SCF1*::*HYGR*	*SCF1* overexpression in IV.1	This study
*WOR2^HA^ P_ADH1_SCF1*	IV.1 *WOR2^HA^*::*SAT1 P_ADH1_SCF1*::*HYGR*	*SCF1* overexpression in *WOR2^HA^* background in IV.1	This study
*wor2*∆/*scf1*∆	IV.1 *wor2*Δ::*HYGR*/*scf1*Δ::*BleMX*	*WOR2*/*SCF1* deletion in IV.1	This study
*wor2*∆/*als4112*∆	IV.1 *wor2*Δ::*HYGR*/*als4112*Δ::*NATR*	*WOR2*/*ALS4112* deletion in IV.1	This study
*wor2*∆/*scf1*∆/*als4112*∆	IV.1 *wor2*Δ::*HYGR*/*scf1*Δ::*BleMX*/*als4112*Δ::*NATR*	*WOR2*/*SCF1*/*ALS4112* deletion in IV.1	This study
*WOR2* ^trunc^	IV.1 *WOR2*^Q354*^ ::*NATR*	*WOR2* truncated genotype in IV.1	This study
*WOR2* ^split^	IV.1 *WOR2*^Q192*^ ::*NATR*	*WOR2* split genotype in IV.1	This study
I.1 *wor2*∆	I.1 *wor2*Δ::*HYGR*	*WOR2* deletion in I.1	This study
II.1 *wor2*∆	II.1 *wor2*Δ::*HYGR*	*WOR2* deletion in II.1	This study
III.8 *wor2*∆	III.8 *wor2*Δ::*HYGR*	*WOR2* deletion in III.8	This study

### Genetic transformations

Details of all genetic constructions are provided in Fig. S6 to S13. Wor2 hyperactivation was achieved by cloning *WOR2* in plasmid pjli8 under the control of the *ADH1* promoter (*P_ADH1_*) with a 3×HA tag at its C-terminus, as previously described (22). The constructs for deletion strains were obtained by fusion PCR using the selection markers *HygR* (for *WOR2* deletion), *NatR* (for *ALS4112* deletion), and *BleMX* (for *SCF1* deletion), flanked by approximately 500 bp upstream and downstream sequences of the target gene. Constructs for Als4112 and Scf1 overexpression were obtained by fusion PCR of an approximately 500 bp upstream sequence of the target gene, the selection marker (*BleMX* or *HygR*, respectively), the *ADH1* promoter, and an approximately 1000 bp sequence of the proximal region of the target gene. Substitution of the *WOR2* full-length genotype by the truncated and split genotypes (*WOR2*^trunc^ and *WOR2*^split^, respectively) was also generated by fusion PCR, including the mutated genotypes and the *NatR* cassette.

Transformation was performed by electroporation and using our CRISPR-Cas9 genome editing protocol, as previously described (46). RNA guides are described in Table S2. Transformants were selected at 37°C on YEPD containing 200 µg/mL of nourseothricin (Werner BioAgents), 800 µg/mL of zeocin (Thermo Fisher Scientific), or 600 µg/mL of hygromycin B (Corning, Corning, NY) according to the selection marker. Correct integration of the constructs was verified by sequencing.

### Transcriptomic analyses

RNA sequencing was performed according to our previously described protocol (21). In brief, RNA was extracted and purified after overnight growth in liquid YEPD (triplicates for each strain). After verification of RNA quality (Fragment Analyzer, Agilent Technologies, Santa Clara, CA), RNA-seq libraries were prepared with the Illumina Stranded mRNA Prep reagents (Illumina, San Diego, CA) and quantified (QubIT, Life Technologies). Sequencing was performed on Illumina NovaSeq 6000 and analyzed using the bcl2fastq2 Conversion Software (version 2.20, Illumina). Reads were aligned to the *C. auris* genome using CLC Genomic Workbench (version 23).

GO term enrichment analysis was performed to group up- and downregulated genes (i.e., ≥2-fold expression change and *P* < 0.05) according to their functionality, as previously described (21).

### Real-time RT-PCR

For analyses in planktonic conditions, strains were grown overnight in liquid YEPD and adjusted to an optical density (OD) of 0.2 in 5 mL of fresh YEPD, followed by an additional 2 h of growth to reach an OD of 0.4. Then, the cultures were centrifuged for 5 min at 2,500 rpm, and the pellet was collected.

For analyses in biofilm conditions, overnight cultures were adjusted to an OD₆₀₀ of 0.27 in 20 mL of RPMI medium and grown in tissue culture flasks (Thermo Fisher Scientific, T75, ref. 156800) for 90 min at 37°C. Planktonic cells were removed by washing with PBS, and 20 mL of fresh RPMI medium was added. After 24 h of incubation at 37°C, biofilms were gently scraped, and the biomass was collected by centrifugation at 2,500 rpm for 5 min.

RNA extraction was performed using the Quick-RNA Fungal/Bacterial Miniprep Kit (ZymoResearch, Lucerna-Chem AG). RNA was purified using a DNA-free kit (Thermo Fisher), and cDNA was synthesized (PrimeScript 1st strand cDNA synthesis kit, Takara Bio). RT-qPCR was performed in biological triplicates and technical duplicates, according to the previously described protocol (22), using primers described in Table S3. Relative expression of the target genes was calculated with the 2^-ΔΔCT^ method and normalized to *ACT1* expression. Statistical analyses were performed using the unpaired *t*-test, and results were considered statistically significant if *P*-value was ≤0.05.

### Crystal violet assay

Measurement of the biofilm mass was performed according to a previously described protocol (21) with some adjustments. Strains grown overnight in liquid YEPD were washed with phosphate buffer saline (PBS) and resuspended in Roswell Park Memorial Institute (RPMI) medium at an OD of 10^6^ cells/mL. Then, 200 µL of these suspensions was added to the wells of a flat-bottomed, polystyrene, untreated Costar 96-well plate (Corning Inc., Corning, NY) for 1.5 h incubation at 37°C without agitation. Unattached cells were next washed with PBS and resuspended in RPMI. The plate was incubated at 37°C, and quantification of biofilm was performed at different time points (6 h, 12 h, 24 h, and 48 h). Then, after two PBS washes, 50 µL of crystal violet 0.5% was added to each well, and the plate was incubated for 45 min at room temperature. The wells were then washed four times with PBS, and 150 µL of ethanol 95% was added for an additional 45 min of incubation. Then, 100 µL was transferred to clean wells, and the absorbance (590 nm) was measured. The experiment was performed in technical triplicates and biological duplicates for each strain. Mean absorbances (representing the biomass of the biofilm) of the different strains were compared using the unpaired *t*-test. For the initial experiment, biofilm quantification was performed at different time points. Based on these results, the optimal time point (i.e., 24 h) was chosen for subsequent experiments.

### Adhesion assay on inert surfaces by flow cytometry

Adhesion of the *C. auris* strains stained with Vybrant DyeCycle Green Stain (Thermo Fisher Scientific) to fluorescent polystyrene microspheres (FluoSpheres Carboxylate-Modified Microspheres, Thermo Fisher Scientific) was assessed by flow cytometry using CytoFlex LX Flow Cytometer (Beckman Coulter Diagnostics), as previously described (21). Yeast cells were detected by their emission within the green fluorescence spectrum ((λem ≈ 488 nm) and further analyzed for adherence to the microspheres in the red fluorescence spectrum (λem ≈ 645 nm) to assess the mean proportions of adherent cells. Results of different strains were compared by the unpaired *t*-test.

### Adhesion assay with keratinocytes

N/TERT-1 human keratinocytes were maintained in keratinocyte-serum-free medium (K-SFM, Gibco) supplemented with bovine pituitary extract (30 µg/mL) and recombinant human epidermal growth factor (0.2 ng/mL), without antibiotics, at 37°C and 5% CO_2_. When 70–90% confluency was reached, cells were rinsed with PBS and detached by addition of 0.05% Trypsin-EDTA. For testing adhesion of *C. auris* to N/TERT-1 keratinocytes, 1.3 × 10^6^ N/TERT-1 cells/well in 2 mL K-SFM medium were seeded in 6-well plates 24 h before infection. On the day of the infection, cell culture media was removed, and 100 yeast cells/well in 1 mL fresh K-SFM medium were added. After 1 h of co-incubation at 37°C and 5% CO_2_, adherent and non-adherent *C. auris* cells were quantified. For quantification of non-adherent cells, the supernatants were collected, and the cells were washed with PBS to ensure complete removal of loosely adherent fungal cells. The supernatants and the wash solution were then pooled and plated on YPD agar. Adherent yeast cells were quantified by adding 2 mL/well melted YPD agar cooled to 56°C. Colonies were enumerated after 1–2 days of incubation at 30°C. The percentage of adherent *C. auris* per well was calculated with the following formula:


$$Adhesion\;\%=\frac{adherent\;CFU}{adherent\;CFU+non\;adherent\;CFU}\times 100$$

### Comparison of *WOR2* sequences from different strains

*WOR2* sequences (1.5 kb, including the entire ORF and flanking regions) from 651 *C. auris* strains were extracted from FungiDB (https://fungidb.org/fungidb/app) and compared with each other. Sequences and alignment are provided in File S2 and S3. Clade assignment was performed by alignments and clustering of the sequences using Geneious Prime, with MAFFT and FastTree implemented in the software. Clades I and III could not be separated in the tree analysis.

## Data Availability

Data are available in the Bioproject PRJNA1332928.

## SUPPLEMENTAL MATERIAL

Supplemental Tables msphere.00057-26-s0001.docx Tables S1 to S3.

Supplemental Figures msphere.00057-26-s0002.pdf Figures S1 to S13.

File S1 msphere.00057-26-s0003.xlsx Raw data of RNA sequencing.

File S2 msphere.00057-26-s0004.xlsx Comparison of *WOR2* sequences from different strains.

File S3 msphere.00057-26-s0005.fasta Comparison of *WOR2* sequences from different strains.

Legends msphere.00057-26-s0006.docx Legends for Files S1 to S3.

ASM does not own the copyrights to Supplemental Material that may be linked to, or accessed through, an article. The authors have granted ASM a non-exclusive, world-wide license to publish the Supplemental Material files. Please contact the corresponding author directly for reuse.
