# Supplementary material for: Role of the transcription factor Wor2 in biofilm formation of Candidozyma auris
Source: mSphere. 2026 Apr 20;11(5):e00057-26. doi: 10.1128/msphere.00057-26 (PMC13203962; doi:10.1128/msphere.00057-26)
Supplement: Supplemental Tables — Tables S1 to S3. [file msphere.00057-26-s0001.docx]

**Table S1. List of the primers used for this study**

| Name of the primer | Sequence of the primer (5’ – 3’) |
| --- | --- |
| *WOR2*_PF_MluI | 5’-ACA CTA CGC GTA TGC CCA AGC TTC CTT CGG T-3’ |
| *WOR2*_PR_BsrGI | 5’-ACA CTT GTA CAT TGG GAG TTG TAA ACC GCC G-3’ |
| HygR FW | 5'-CCT TTC TGC CTA GCT CCT TCG TAT AGT GCT TGC TGT TCG ATA-3' |
| HygR REV | 5'-GAC AGA CAG TCA GCC AAC TCT ATT TTA TGA TGG AAT GAA TGG GAT G-3' |
| *WOR2* up FW | 5'-AGC TCT ATC ATT TCA TCG CCC TTT-3' |
| *WOR2* up REV | 5'-TAT CGA ACA GCA AGC ACT ATA CGA AGG AGC TAG GCA GAA AGG-3' |
| *WOR2* down FW | 5'-CAT CCC ATT CAT TCC ATC ATA AAA TAG AGT TGG CTG ACT GTC TGT C-3' |
| *WOR2* down REV | 5'-GGA GTA ATG AGT CAG CCG GA-3' |
| Fusion PCR finale del *WOR2* FW | 5'-TTC TGG CGA CCG CTT GCC-3' |
| Fusion PCR finale del *WOR2* REV | 5'-TGG AGT TTG GTA ATT ACC GGC TC-3' |
| P*_ADH1_* FW | 5'-CGA GAT AGA TCG AAA TAC GCT CTA A-3' |
| P*_ADH1_* REV | 5'-GCA GCA AGC GAA GCA AGT TTC ATG ATT TCG TGA AGA TTG ATT GAT GAT GG-3' |
| ZeoR FW | 5'-TTC ACC TTC TTT CCC ATC ATC TTT CGG CGC GCC AGA TCT GTT TA-3' |
| ZeoR REV | 5'-TAA ACA GAT CTG GCG CGC CGA AAG ATG ATG GGA AAG AAG GTG AA-3' |
| 5’ *ALS4112* FW | 5'-ACC ACG AGA CAA AAG CTG CCA-3' |
| 5’ *ALS4112* REV | 5'-TTA GAG CGT ATT TCG ATC TAT CTC GTC ATT AAT GCA GGT TAA CCT GGC TT-3' |
| *ALS4112* FW | 5'-CCA TCA TCA ATC AAT CTT CAC GAA ATC ATG AAA CTT GCT TCG CTT GCT GC-3' |
| *ALS4112* REV | 5'-TCA GAA CAT GAG GTG GAA AAG TGG-3' |
| Fusion PCR finale *ALS4112* FW | 5'-ACG ATT TAA AAC GCA TAA GGT GGT C-3' |
| Fusion PCR finale *ALS4112* REV | 5'-GCA ACA ATA CCA GAG ATT TTG ACA G-3' |
| P*_ADH1_* FW | 5'-CGA GAT AGA TCG AAA TAC GCT CTA A-3' |
| P*_ADH1_* REV | 5'-CTG CAA GAA GTA AAG AGA ATC TCA TGA TTT CGT GAA GAT TGA TTG ATG ATG G-3' |
| HygR FW | 5'-CTC TAT CTT AAA ACT TCA CCT CCA CAG TAT AGT GCT TGC TGT TCG ATA-3' |
| HygR REV | 5'-TTA GAG CGT ATT TCG ATC TAT CTC GAT TTT ATG ATG GAA TGA ATG GGA TG-3' |
| 5’ *SCF1* FW | 5'-GGG CTC GCG GCT TAC AAT G-3' |
| 5’ *SCF1* REV | 5'-TAT CGA ACA GCA AGC ACT ATA CTG TGG AGG TGA AGT TTT AAG ATA GAG-3' |
| *SCF1* FW | 5'-CCA TCA TCA ATC AAT CTT CAC GAA ATC ATG AGA TTC TCT TTA CTT CTT GCA G-3' |
| *SCF1* REV | 5'-CTG GTC TCA GTA GAC GAG GTT-3' |
| Fusion PCR finale *SCF1* FW | 5'-GCG TGC CAT TGA CAC AGG AA-3' |
| Fusion PCR finale *SCF1* REV | 5'-GGA AGT AGT CTC AGT GCT GGT-3' |
| NatR FW | 5'-TTC ACC TTC TTT CCC ATC ATC TTT CGA CAT GGA GGC CCA GAA TAC-3' |
| NatR REV | 5'-AAG CAG GTA TCA TAA AAG CTC ACG TCA GTA TAG CGA CCA GCA TTC AC-3' |
| *ALS4112* up FW | 5'-GTA TTC TGG GCC TCC ATG TCG AAA GAT GAT GGG AAA GAA GGT GAA-3' |
| *ALS4112* up REV | 5‘-GTATTCTGGGCCTCCATGTCGAAAGATGATGGGAAAGAAGGTGAA-3‘ |
| *ALS4112* down FW | 5'-GTG AAT GCT GGT CGC TAT ACT GAC GTG AGC TTT TAT GAT ACC TGC TT-3' |
| *ALS4112* down REV | 5‘-CCCTCGTATTTGCCAGTCCATT-3‘ |
| Fusion PCR finale del *ALS4112* FW | 5’-TGGCATTCGTTCATATCCACATGTA-3’ |
| Fusion PCR finale del *ALS4112* REV | 5’-AAATTGTGAAAGAAGAGCACCACAC-3’ |
| ZeoR FW | 5'-CTC TAT CTT AAA ACT TCA CCT CCA CGG CGC GCC AGA TCT GTT TA-3' |
| ZeoR REV | 5'-CAC TAG GTC CAC TTG GTC CTT CAT TAA TGC AGG TTA ACC TGG CTT-3' |
| *SCF1* up FW | 5’-CGGCTTACAATGCCCAGAATG-3’ |
| *SCF1* up REV | 5'-TAA ACA GAT CTG GCG CGC CGT GGA GGT GAA GTT TTA AGA TAG AG-3' |
| *SCF1* down FW | 5'-AAG CCA GGT TAA CCT GCA TTA ATG AAG GAC CAA GTG GAC CTA GTG-3' |
| *SCF1* down REV | 5’-TGAGATACACTAGCAGAGGGTC-3’ |
| Fusion PCR finale del *SCF1* FW | 5’-CACAGGAAGAACCACTTTCACG-3’ |
| Fusion PCR finale del *SCF1* REV | 5’-AACTTGGGAGGCAGAAGGAG-3’ |
| 1 FW | 5'-TCG ACC GCT CTC CTA ACC AC-3' |
| 1 REV | 5'-GAA GCG TAG GCC GCT CCT AGA CCG CTG CCC GCT C-3' |
| 2 FW | 5'-GAG CGG GCA GCG GTC TAG GAG CGG CCT ACG CTT C-3' |
| 2 REV | 5'-GTA TTC TGG GCC TCC ATG TCT CAA CGC AGC CCC TTG GC-3' |
| 3 FW | 5'-GCC AAG GGG CTG CGT TGA GAC ATG GAG GCC CAG AAT AC-3' |
| 3 REV | 5'-GGC TCT GTT TGC GGC GGC AGT ATA GCG ACC AGC ATT CAC-3' |
| 4 FW | 5'-GTG AAT GCT GGT CGC TAT ACT GCC GCC GCA AAC AGA GCC-3' |
| 4 REV | 5'-CGC ACT CGC ACT GAG CAC T-3' |
| Fusion *WOR2* mut FW | 5'-AGC ACC TGG ACT CGA GCC-3' |
| Fusion *WOR2* mut REV | 5'-ACT GGG GCG ACT TAG GTT TCT-3' |
| 1 FW II | 5'-TCC CGG GCC TTT TTC TGG C-3' |
| 1 REV II | 5'-GGC TAG AGC GGA AGT GGA TGT TGT TGA GGT TGT TGT TGT TGC TGT TGT TGT TGG AT-3' |
| 2 FW II | 5'-ATC CAA CAA CAA CAG CAA CAA CAA CAA CCT CAA CAA CAT CCA CTT CCG CTC TAG CC-3' |
| Fusion *WOR2* II FW | 5'-CCG TTC ATG GCC TGT ACG C-3' |

**Table S2. List of the ARN guides used for this study**

| **Name of the ARN guide** | **Description** | **Sequence of the ARN guide (5’ – 3’)** |
| --- | --- | --- |
| CauNi_sg5’ | Guide RNA for *WOR2* hyperactivation | CCCGGAGAUACACGGCGCCG |
| CauNi_sg3’ | Guide RNA for *WOR2* hyperactivation | GCUGCAAAAUAAGGCCAGAG |
| RNAg *WOR2*del 5’ | Guide RNA for *WOR2* deletion | UCGGUGUCUGAGUUGAUUGU |
| RNAg *WOR2*del 3’ | Guide RNA for *WOR2* deletion | ACGCUUCGUGAGCUGGCGGC |
| RNAg *ALS4112*-OE | Guide RNA for *ALS4112* overexpression | GAUGAUGGGAAAGAAGGUGA |
| RNAg SCF*1*-OE | Guide RNA for *SCF1* overexpression | ACUUGCCGUAUAUAAAACCA |
| RNAg *ALS4112*del 5’ | Guide RNA for *ALS4112* deletion | ACCCAGCUGCAACUUAGCGA |
| RNAg *ALS4112*del 3’ | Guide RNA for *ALS4112* deletion | CAGGGUAUGUUUGAAUCACA |
| RNAg *SCF1*del 5’ | Guide RNA for *SCF1* deletion | CACUUACACAACAAGCCCAU |
| RNAg *SCF1*del 3’ | Guide RNA for *SCF1* deletion | UAGUGGACCACAAGGACCAG |
| RNAg *WOR2*mut | Guide RNA for *WOR2*^trunc^ & *WOR2*^split^ strains | CAGCACGUGAGCAGGUAAGG |

**Table S3. List of the primers for RT-qPCR used for this study**

| **Name of the primer** | **Sequence of the primer (5’ – 3’)** |
| --- | --- |
| *YWP1* qPCR FW | CCAGCCCCAACTTCTCCAG |
| *YWP1* qPCR REV | GAGAGGAAGAGCAGCCATGG |
| *ALS4112* qPCR FW | GGTCCCTCTGTCACACCTCA |
| *ALS4112* qPCR REV | CCCTTGTAGCAAACCTCAGAG |
| *SCF1* qPCR FW | AGCACTGAACCACCTTCTGTTAC |
| *SCF1* qPCR REV | TTCTGTGCCAGTACTCGTGTTTG |
| *WOR2* qPCR FW | GACGAGCGGAAACCACATTG |
| *WOR2* qPCR REV | GTACTCCGGGTCCTTGTGTG |
| *WOR2*-a II qPCR FW | AACGCCTCTGTATGAGCAACCT |
| *WOR2*-a II qPCR REV | GTTGCTGTTGTTGTTGGATATGAG |
