# Supplementary material for: Role of the transcription factor Wor2 in biofilm formation of Candidozyma auris
Source: mSphere. 2026 Apr 20;11(5):e00057-26. doi: 10.1128/msphere.00057-26 (PMC13203962; doi:10.1128/msphere.00057-26)
Supplement: Supplemental Figures — Figures S1 to S13. [file msphere.00057-26-s0002.pdf]

Figure S1

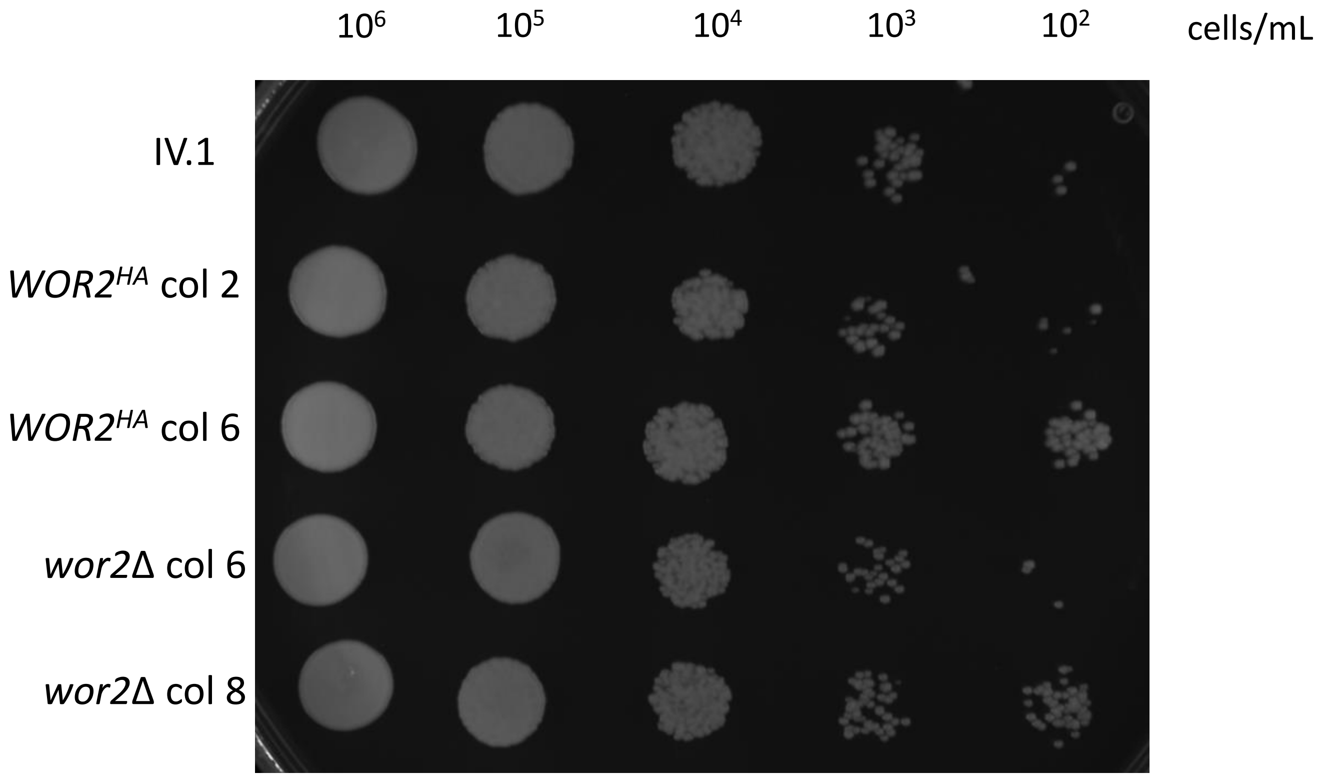

Growth comparison between strains IV.1, *WOR2^{HA}* and *wor2*Δ.

Different inocula (10^2 to 10^6 cells/mL) of strains IV.1, *WOR2^{HA}* (colonies 2 and 6) and *wor2*Δ (colonies 6 and 8) were spotted on YEPD agar. Pictures were taken after 24h growth at 37°C.

Figure S2

RT 30°C 37°C 42°C

IV.1

WOR2^{HA}

wor2Δ

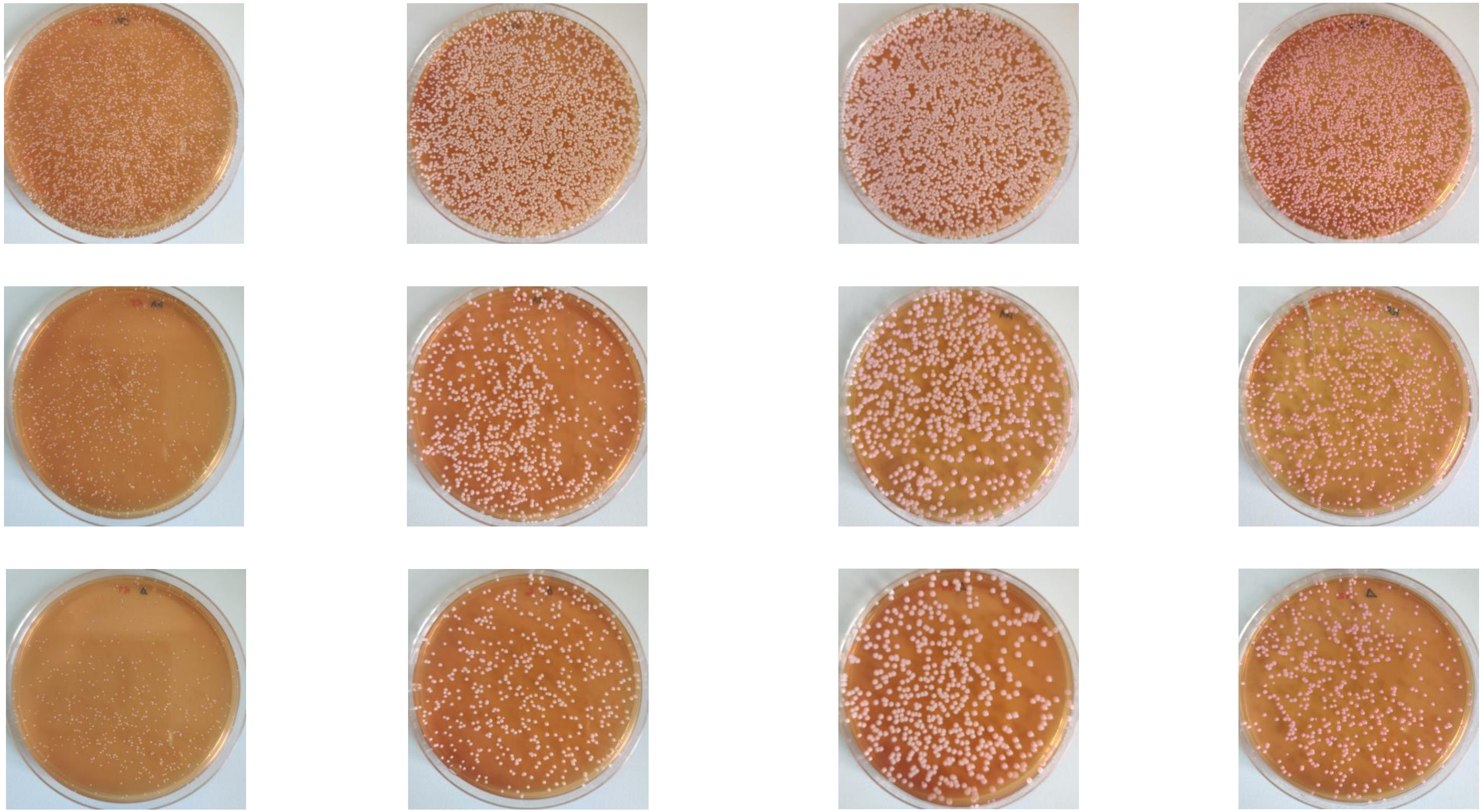

Phenotypic comparison of IV.1, *WOR2^{HA}* and *wor2Δ* at different temperatures with addition of phloxine B (5 µg/mL).
Cells were diluted into PBS to reach a concentration of 500 cells/mL and spred wih glass beads on YPD agar plates with 5 µg/mL of phloxine B (an agent known to stain in pink colonies in opaque-state). Growth at different temperature was tested : room temperature (RT), 30°C, 37°C and 42°C. Pictures were taken after 48h growth.

Figure S3

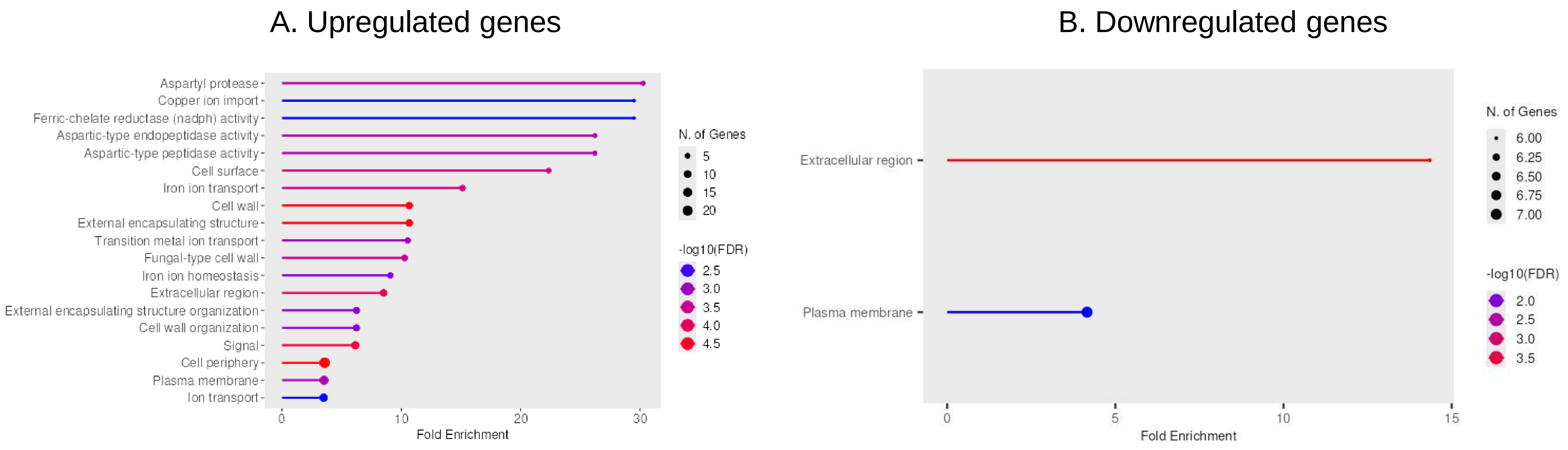

Gene ontology (GO) analysis of upregulated genes (A) and downregulated genes (B) in $\textit{WOR2}^{\textit{HA}}$ compared to IV.1 (background strain). The x-axis represents the fold enrichment, which indicates how much more frequently each GO term appears among the analyzed gene set compared to its frequency in the entire genome background. The color gradient represents the $-\log_{10}$(FDR) values, with red and blue corresponding to more and less significant enrichments, respectively. Dot size reflects the number of genes associated with each GO term.

Figure S4

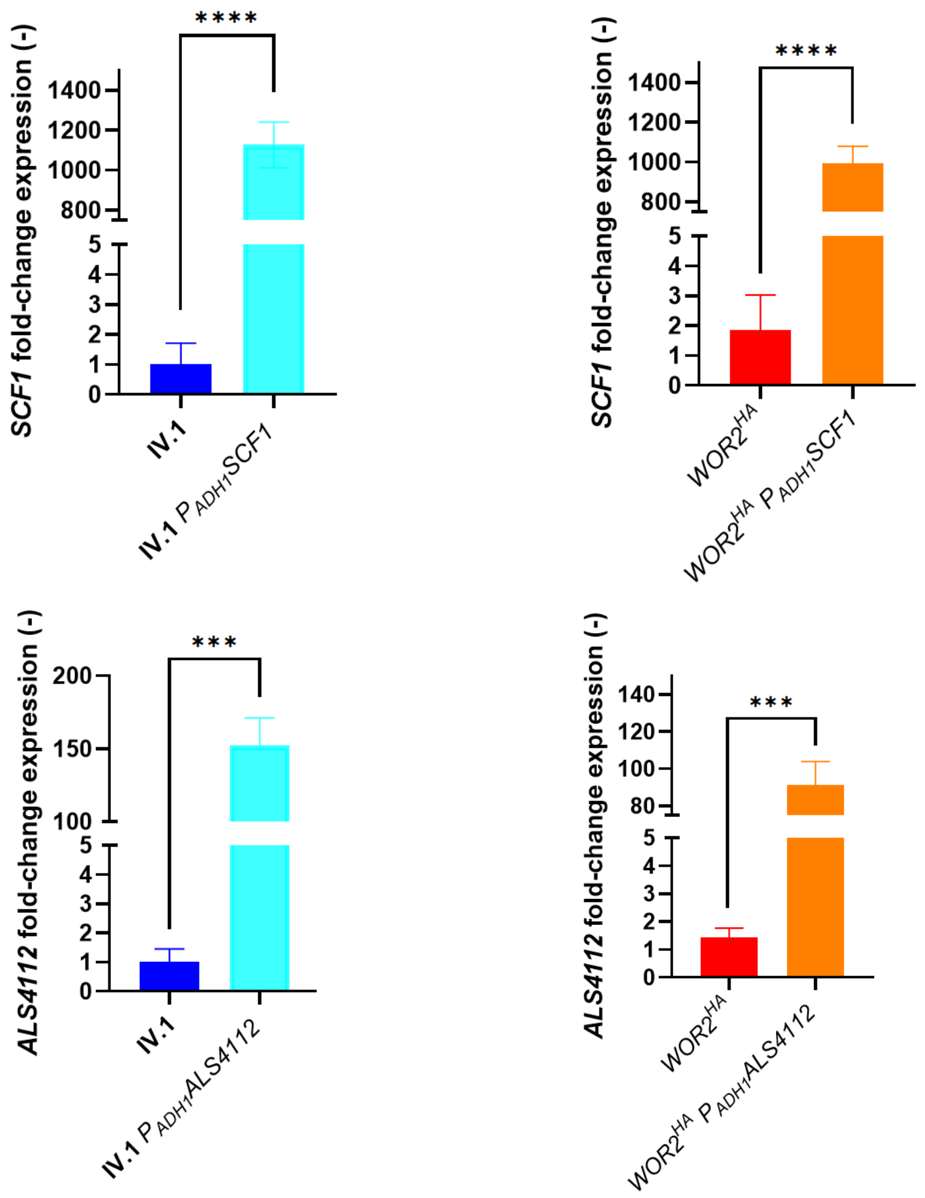

Verification of the overexpression of *ALS4112* and *SCF1* in mutant strains with constitutive expression of *SCF1* and *ALS4112* under the *ADH1* promoter.

Results are expressed as fold-change compared to their respective parental strains. Bars represent means with standard deviations of three biological replicates. Statistical analysis was performed using unpaired t-test with significant p-value defined as ≤ 0.05 (* ≤ 0.05, ** ≤ 0.01, *** ≤ 0.001, **** ≤ 0.0001).

Figure S5

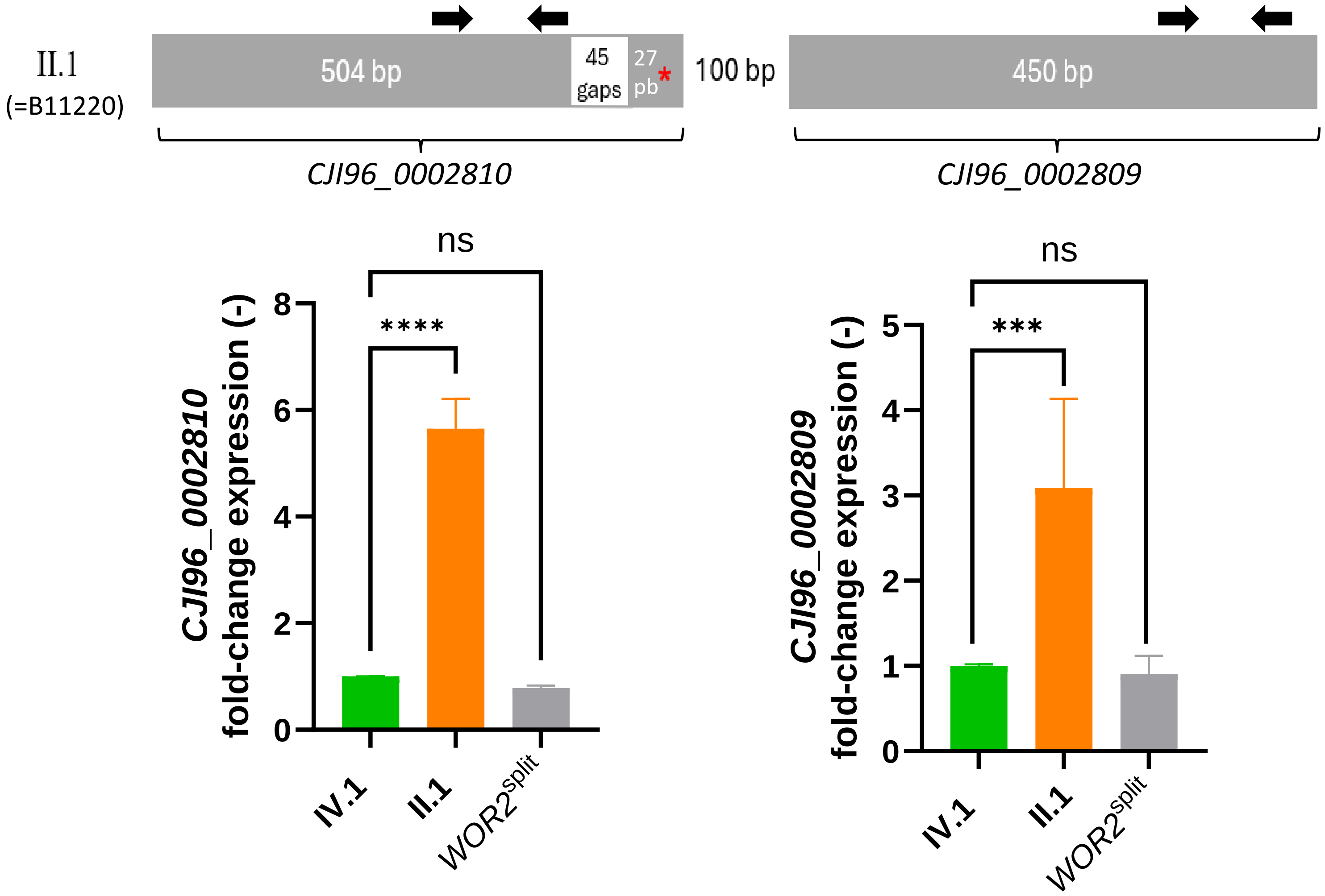

Figure S3. Relative expression of the two *WOR2* ORFs in clade II.

The isolate from clade II (II.1 = B11220) carries a split form of *WOR2*, resulting in two ORFs, *CJI96_0002809* and *CJI96_0002810*. The expression levels of these two loci were measured in isolates IV.1, II.1, and *WOR2*^{split} (strain IV.1 carrying the clade II.1 *WOR2* genotype) by RT-qPCR. The primers used for each RT-qPCR are indicated by black arrows. Results are expressed as fold-change relative to the wild-type IV.1 strain. Bars represent the mean ± standard deviation from biological replicates. Statistical analysis was performed using an unpaired *t*-test, with significance defined as $P \leq 0.05$ (* ≤ 0.05, ** ≤ 0.01, *** ≤ 0.001, **** ≤ 0.0001).

Figure S6

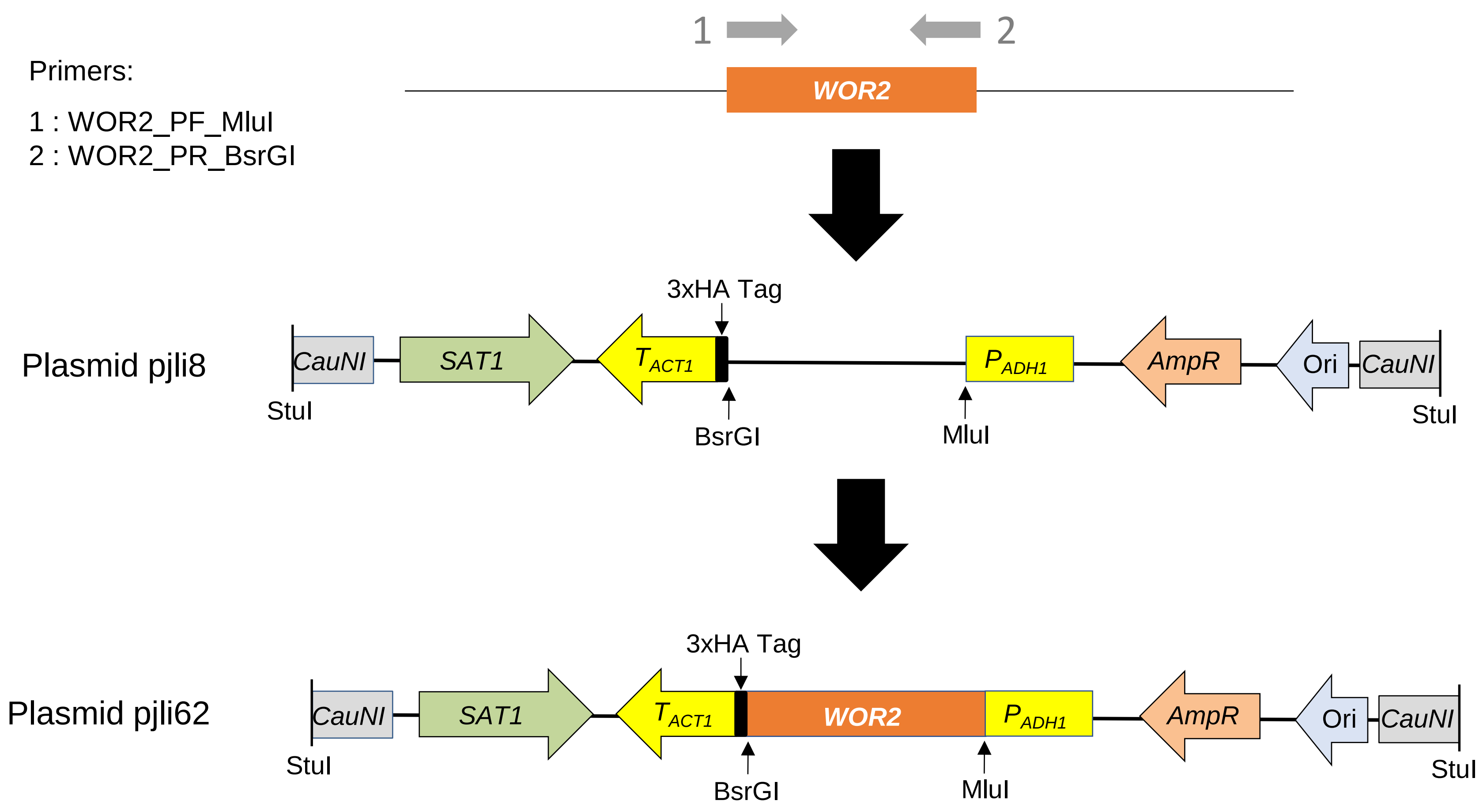

Construct for Wor2 hyperactivation (*WOR2*^{HA} strain). The *WOR2* PCR product was cloned at MluI and BsrGI sites in plasmid pjli8 containing the promotor P_{ADH1}, the 3xHa Tag, the terminator T_{ACT1}, the *SAT1* cassette (nourseothricin resistance) and the *C. auris* neutral site *CauNI*. The resulting plasmid was linearized by StuI.

Figure S7

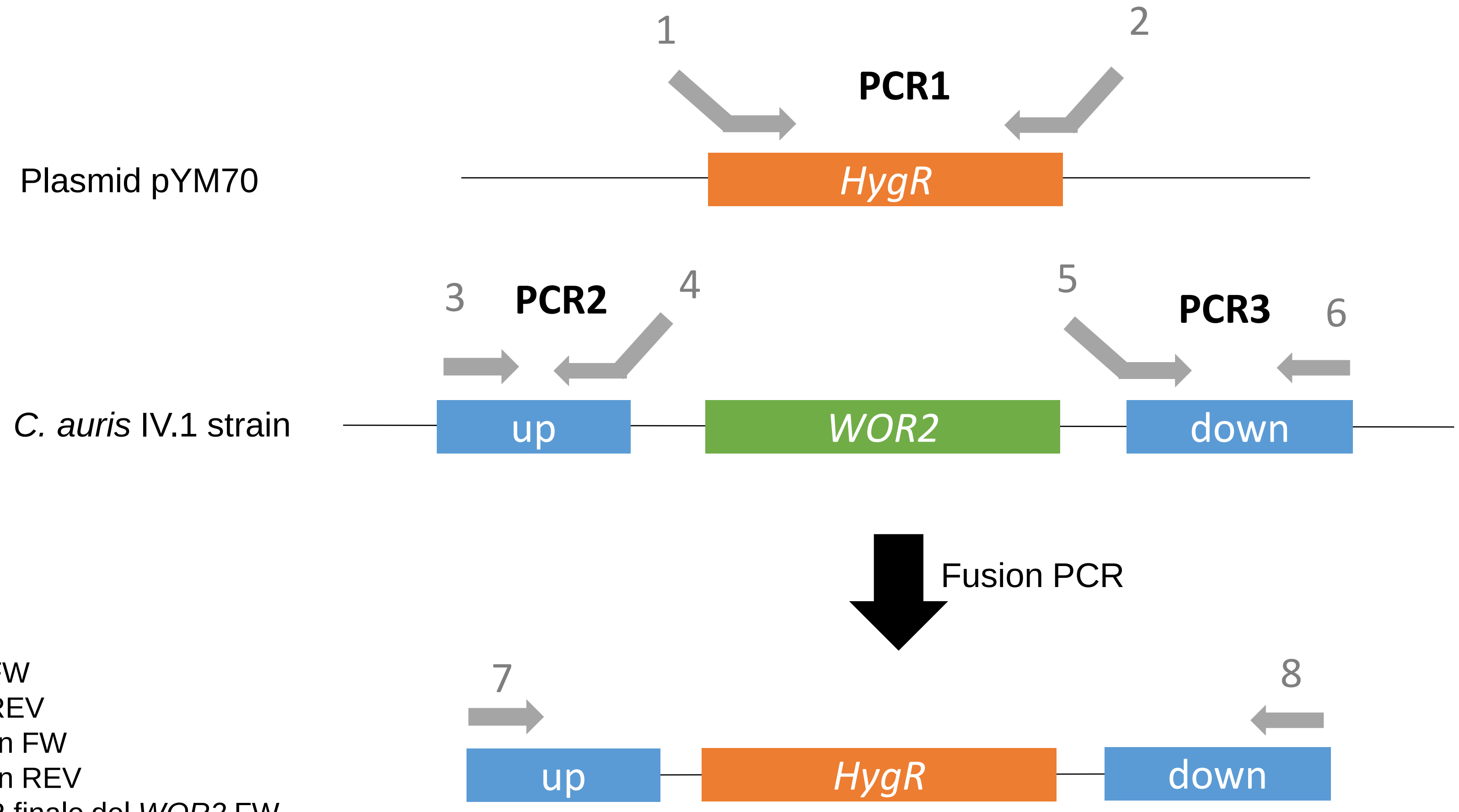

Primers:

1 : HygR FW
2 : HygR REV
3 : *WOR2* up FW
4 : *WOR2* up REV
5 : *WOR2* down FW
6 : *WOR2* down REV
7 : Fusion PCR finale del *WOR2* FW
8 : Fusion PCR finale del *WOR2* REV

Construct for *WOR2* deletion (*wor2*Δ strain). The construct was obtained by fusion PCR of an approximately 500 bp upstream region (up) of *WOR2*, the *HygR* cassette and an approximately 500 bp downstream region (down) of *WOR2*.

Figure S8

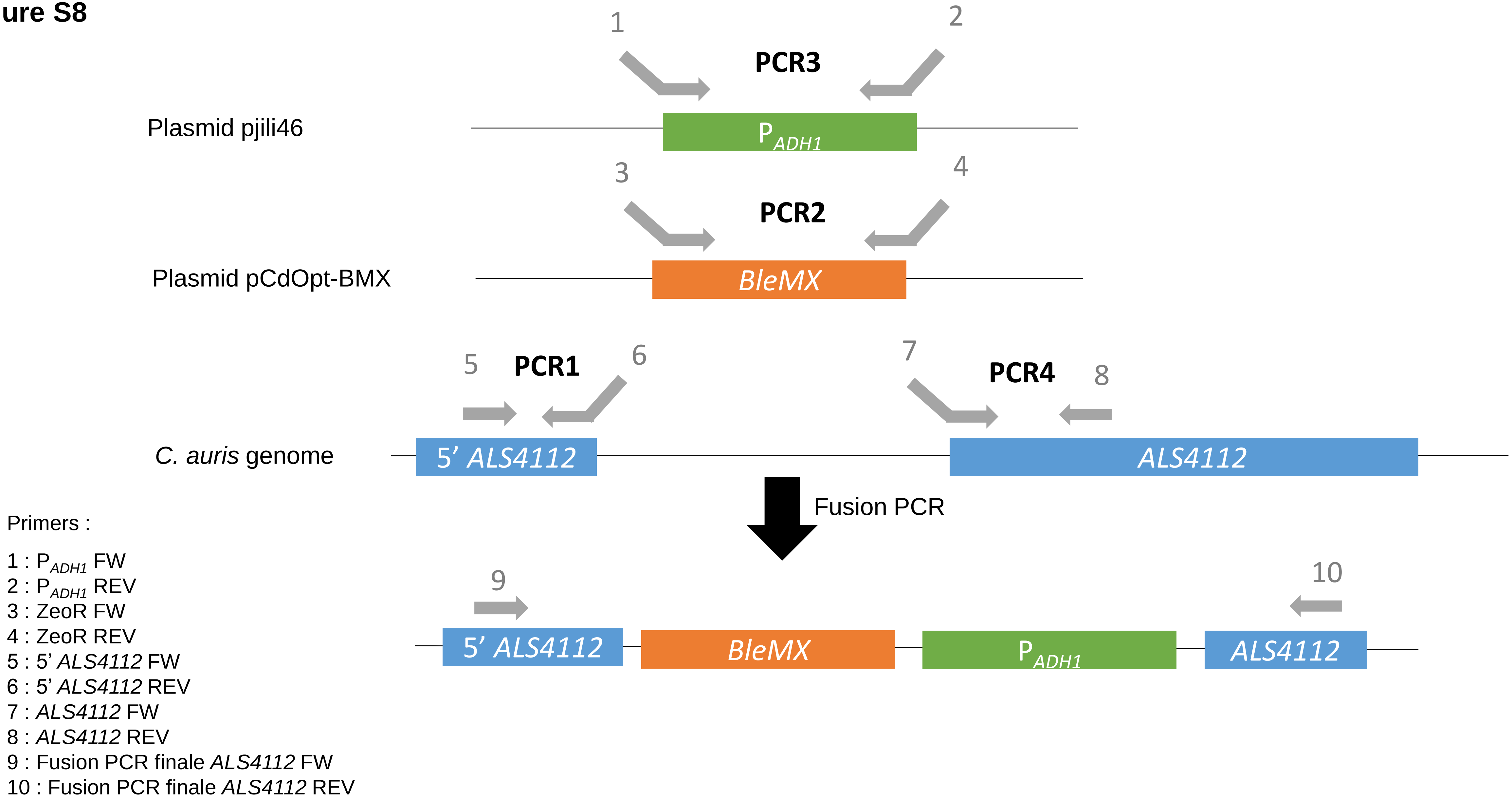

Construct for *ALS4112* overexpression (P_{ADH1}*ALS4112* and *WOR2*^{HA} P_{ADH1}*ALS4112* strains). The construct was obtained by fusion PCR of an approximately 500 bp upstream region of *ALS4112* (5’ *ALS4112*), the *BleMX* cassette, the *ADH1* promoter and the proximal sequence (approximately 1000 bp) of *ALS4112*.

Figure S9

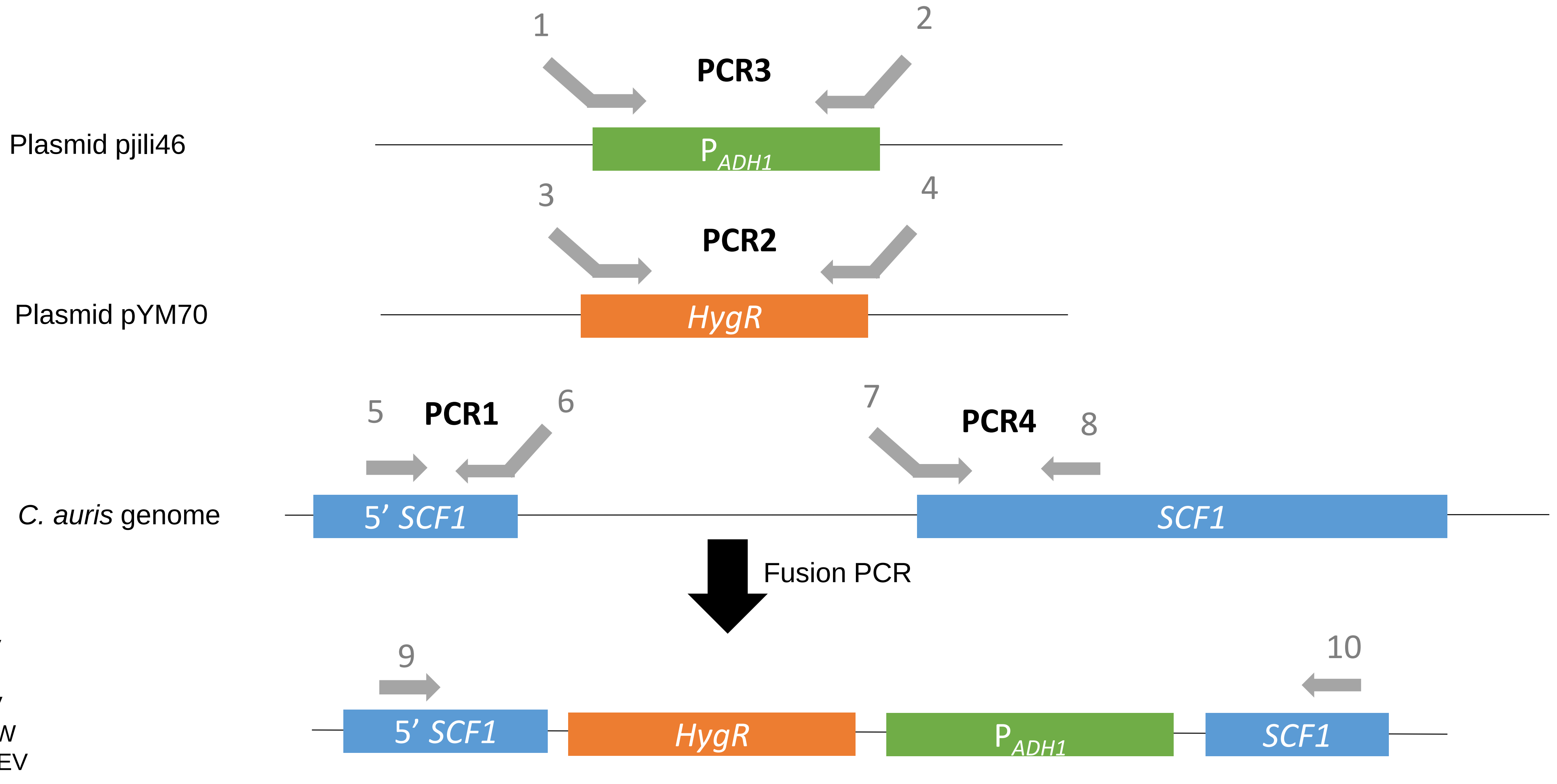

Primers :

1 : P_{ADH1} FW
2 : P_{ADH1} REV
3 : HygR FW
4 : HygR REV
5 : 5’ *SCF1* FW
6 : 5’ *SCF1* REV
7 : *SCF1* FW
8 : *SCF1* REV
9 : Fusion PCR finale *SCF1* FW
10 : Fusion PCR finale *SCF1* REV

Construct for *SCF1* overexpression (P_{ADH1}*SCF1* and *WOR2*^{HA} P_{ADH1}*SCF1* strains). The construct was obtained by fusion PCR of an approximately 500 bp upstream region of *SCF1* (5’ *SCF1*) , the *HygR* cassette, the *ADH1* promoter and the proximal sequence (approximately 1000 bp) of *SCF1*.

Figure S10

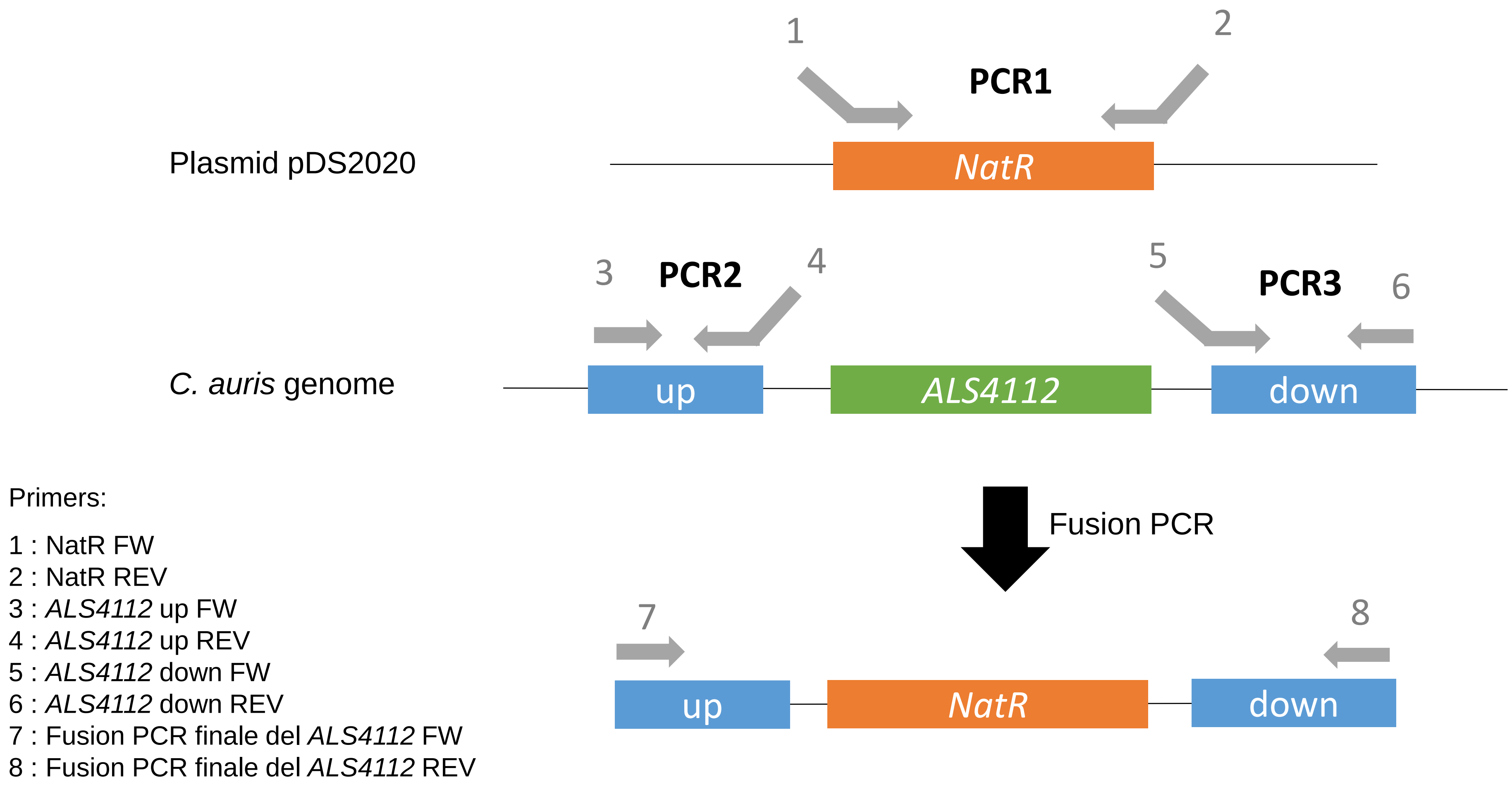

Construct for *ALS4112* deletion in wor2Δ background (*wor2Δ als4112Δ* and *wor2Δ scf1Δ als4112Δ* strains). The construct was obtained by fusion PCR of an approximately 500 bp upstream region (up) of *ALS4112*, the *NatR* cassette and an approximately 500 bp downstream region (down) of *ALS4112*.

Figure S11

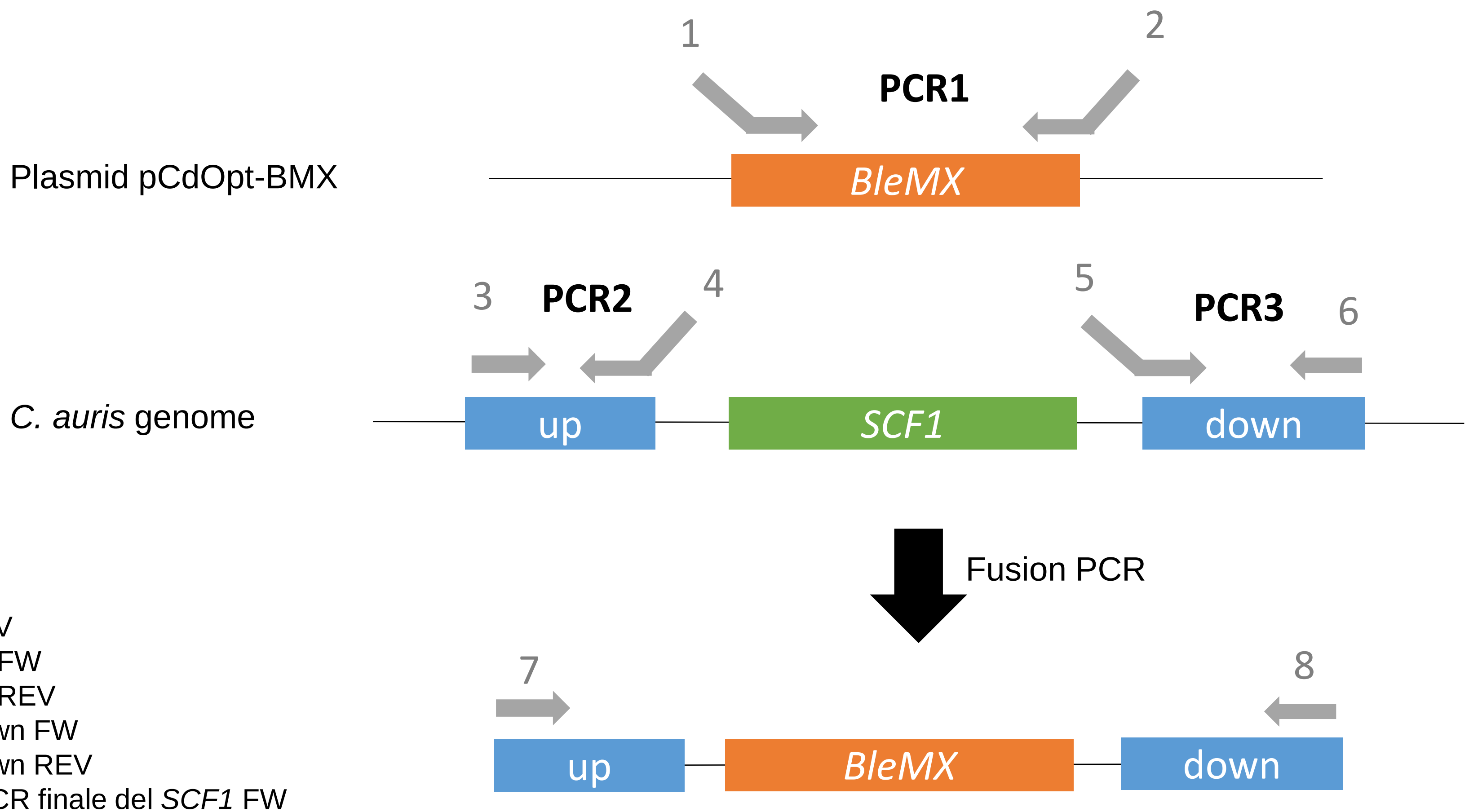

Primers:

1 : ZeoR FW
2 : ZeoR REV
3 : *SCF1* up FW
4 : *SCF1* up REV
5 : *SCF1* down FW
6 : *SCF1* down REV
7 : Fusion PCR finale del *SCF1* FW
8 : Fusion PCR finale del *SCF1* REV

Construct for *SCF1* deletion in wor2Δ background (*wor2Δ scf1Δ* and *wor2Δ scf1Δ als4112Δ* strains). The construct was obtained by fusion PCR of an approximately 500 bp upstream region (up) of *SCF1*, the *BleMX* cassette and an approximately 500 bp downstream region (down) of *SCF1*.

Figure S12

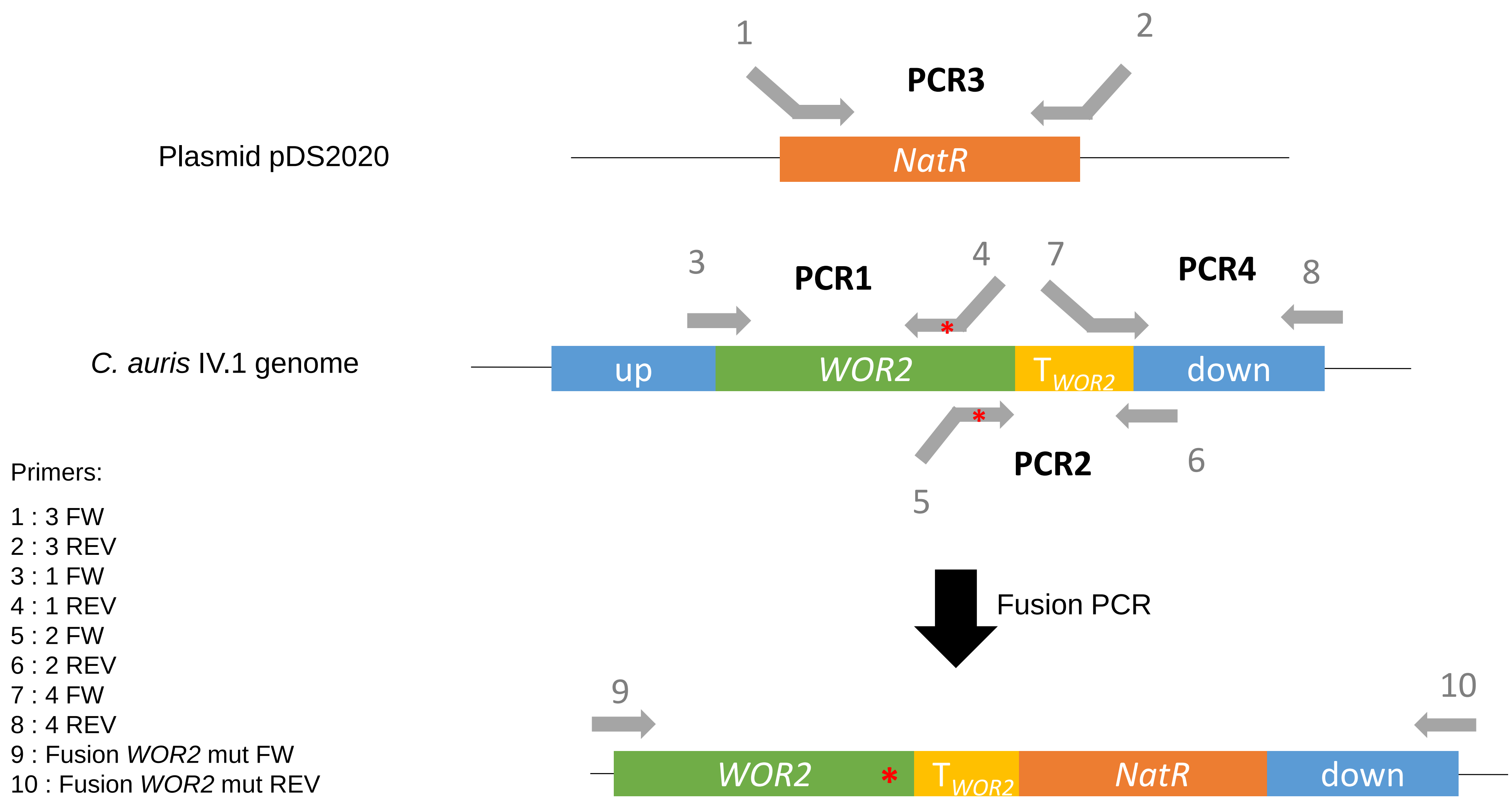

Construct for *WOR2*^{trunc} strain (substitution of *WOR2* wild-type genotype by the "truncated genotype")***.*** The construct was obtained by fusion PCR of the *WOR2* truncated genotype, the *WOR2* terminator (T_{WOR2}), the NatR cassette and an approximately 500 bp downstream region (down) of *WOR2*. The red star indicates the mutation for the stop codon (contained in primers 4 and 5) to generate the truncated genotype.

Figure S13

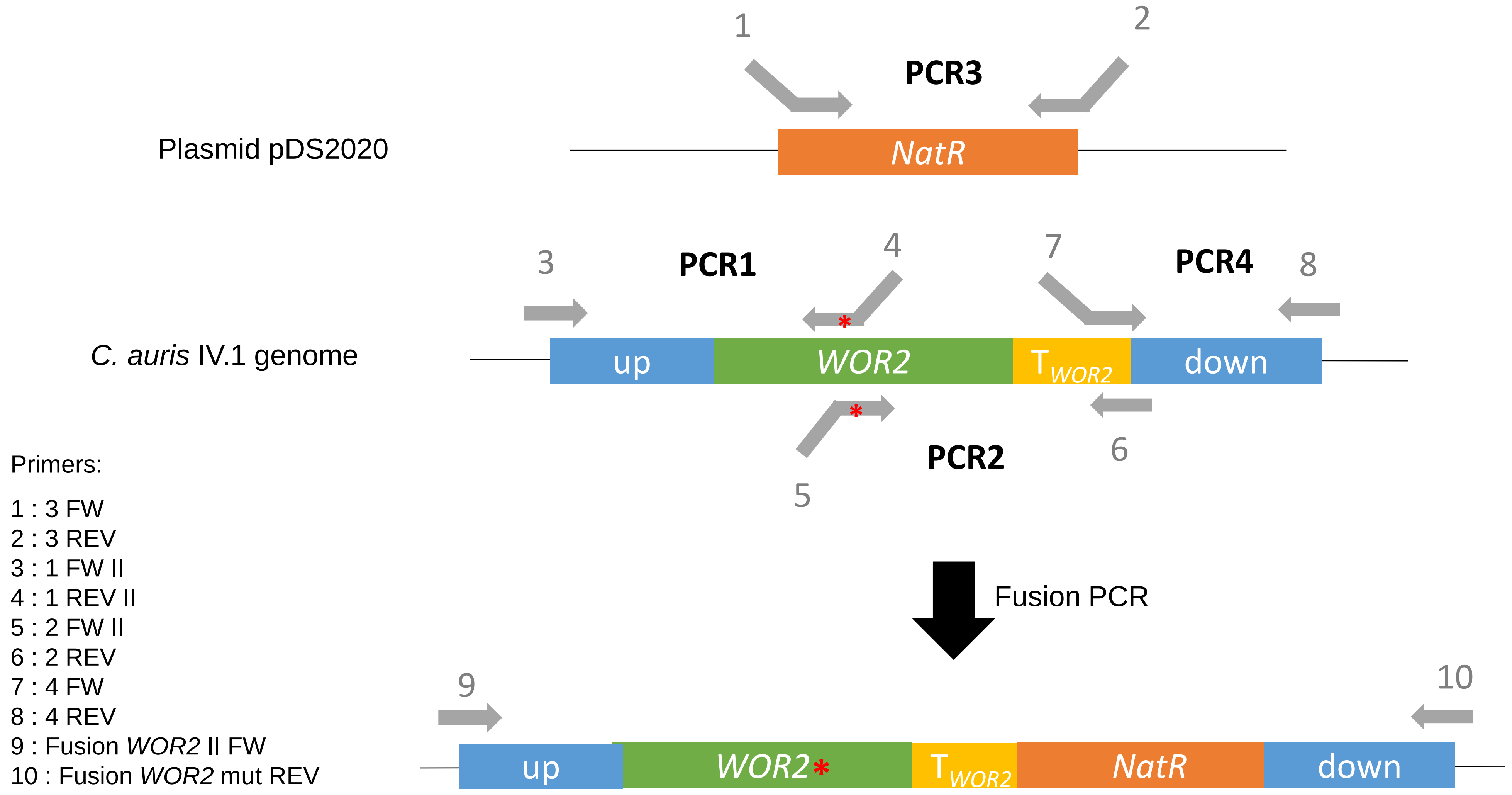

Construct for *WOR2*[split] strain (substitution of *WOR2* wild-type genotype by the "split genotype")**.** The WOR2 split genotype was obtained by fusion a sequence containing an approximately 500 pb upstream region (up) of *WOR2* and the proximal region of the split *WOR2* genotype with a sequence containing the distal region of the split *WOR2* genotype and the *WOR2* terminator (T_{WOR2}). The resulting fusion PCR product was fused tot the NatR cassette and an approximately 500 bp downstream region (down) of *WOR2*.

The red star indicates the mutation for the stop codon and gap (contained in primers 4 and 5) to generate the split genotype.
