# Supplementary material for: Role of the transcription factor Wor2 in biofilm formation of Candidozyma auris
Source: mSphere. 2026 Apr 20;11(5):e00057-26. doi: 10.1128/msphere.00057-26 (PMC13203962; doi:10.1128/msphere.00057-26)
Supplement: Legends — for Files S1 to S3. [file msphere.00057-26-s0006.docx]

**Supplementary Files Legends**

**File S1. Raw data of RNA sequencing for *WOR2*^HA^ and its parental strain IV.1**

Differential gene expression of *WOR2*^HA^ is expressed as fold-change compared to IV.1.

Sheet 1. Differential expression of all genes.

Sheet 2. Genes with significant upregulation (i.e. ≥2-fold expression increase and p<0.05) in *WOR2*^HA^ compared to IV.1.

Sheet 3. Genes with significant downregulation (i.e. ≥2-fold expression decrease and p<0.05) in *WOR2*^HA^ compared to IV.1.

**File S2.** **Comparison of *WOR2* sequences from different strains**

Alignment of *WOR2* sequences (1,5 kb including the entire ORF and flanking regions) of 651 *C. auris* strains represented in excel file.

Sequences were extracted from FungiDB (<https://fungidb.org/fungidb/app>).

**File S3.** **Comparison of *WOR2* sequences from different strains**

Alignment of *WOR2* sequences (1,5 kb including the entire ORF and flanking regions) of 651 *C. auris* strains represented in fasta file.

Sequences were extracted from FungiDB (<https://fungidb.org/fungidb/app>).
